# Longer Asthma Duration Is Associated With Elevated Non‐T2 Sputum Biomarkers and Reduced T2 Inflammation in Severe Asthma

**DOI:** 10.1111/all.70436

**Published:** 2026-07-09

**Authors:** Freda Yang, Sujin Seo, Takehiro Hasegawa, Chloe I. Bloom, Nazanin Zounemat‐Kermani, Pankaj K. Bhavsar, Katie Raby, Ian M. Adcock, Sungho Won, Tae‐Bum Kim, Kian Fan Chung, the Unbiased Biomarkers for the Prediction of Respiratory Disease Outcomes (U‐BIOPRED) Consortium, M. I. Abdel‐Aziz, C. Auffray, Y. E. Badi, P. Bakke, A. T. Bansal, F. Baribaud, S. Bates, A. F. Behndig, E. H. Bel, J. Bigler, B. Billing, M. J. Boedigheimer, K. Bønnelykke, J. Brandsma, P. Brinkman, E. Bucchioni, D. Burg, A. Bush, M. Caruso, R. Chalekis, P. Chanez, B. Chaves, F. K. Chung, T. Checa, C. H. Compton, J. Corfield, D. Cunoosamy, A. D'Amico, B. Dahlén, B. De Meulder, R. Djukanovic, V. J. Erpenbeck, D. Erzen, K. Fichtner, L. J. Fleming, E. Formaggio, S. J. Fowler, U. Frey, M. Gahlemann, T. Geiser, V. Goss, Y. Guo, S. Hashimoto, J. Haughney, G. Hedlin, P. W. Hekking, T. Higenbottam, J. M. Hohlfeld, C. Holweg, I. Horváth, P. Howarth, A. J. James, R. G. Knowles, J. Kolmert, J. Konradsen, N. Krug, N. Lazarinis, C.‐X. Li, M. J. Loza, R. Lutter, A. Manta, S. Masefield, Maitland‐van der Zee Anke‐Hilse, J. G. Matthews, A. Mazein, R. J. M. Middelveld, M. Miralpeix, C. S. Murray, J. Musial, S. Mumby, D. Myles, B. Nordlund, H. Olsson, I. Pandis, S. Pavlidis, A. Platt, A. Postle, P. Powel, G. Praticò, M. Puig Valls, N. Rao, S. Reinke, J. Riley, A. Roberts, G. Roberts, A. Rowe, T. Sandström, J. P. R. Schofield, W. Seibold, D. E. Shaw, R. Sigmund, F. Singer, P. J. Skipp, M. Smicker, A. R. Sousa, M. Sparreman‐Mikus, P. J. Sterk, K. Sun, B. Thornton, A. Versi, N. H. Vissing, A. M. Wheelock, C. E. Wheelock, S. J. Wilson, V. Yasinska, Aliprantis Antonios, Allen David, Alving Kjell, P. Badorrek, Balgoma David, S. Ballereau, Barber Clair, Batuwitage Manohara Kanangana, Bautmans An, A. Bedding, A. F. Behndig, Beleta Jorge, A. Berglind, Bochenek Grazyna, Braun Armin, D. Campagna, Carayannopoulos Leon, C. Casaulta, A. Chaiboonchoe, Chaleckis Romanas, Davison Timothy, De Alba Jorge, Dekker Tamara, Delin Ingrid, P. Dennison, Dijkhuis Annemiek, Dodson Paul, Draper Aleksandra, K. Dyson, Edwards Jessica, L. El Hadjam, Emma Rosalia, C. Faulenbach, Flood Breda, G. Galffy, Gallart Hector, D. Garissi, J. Gent, M. Gerhardsson de Verdier, D. Gibeon, Gomez Cristina, Gove Kerry, Gozzard Neil, E. Guillmant‐Farry, E. Henriksson, Hewitt Lorraine, U. Hoda, Hu Richard, Hu Sile, X. Hu, E. Jeyasingham, K. Johnson, N. Jullian, Kamphuis Juliette, J. Kennington Erika, Kerry Dyson, G. Kerry, M. Klüglich, Knobel Hugo, J. Knox Alan, Kolmert Johan, J. R. Konradsen, Kots Maxim, Kretsos Kosmas, L. Krueger, Kuo Scott, Kupczyk Maciej, A.‐S Lantz, Larminie Christopher, L. X. Larsson, P. Latzin, N. Lazarinis, Lefaudeux Diane, N. Lemonnier, Li Chuan‐Xing, Lone‐Latif Saeeda, L. A. Lowe, Manta Alexander, Marouzet Lisa, Martin Jane, Mathon Caroline, L. McEvoy, Meah Sally, A. Menzies‐Gow, Metcalf Leanne, Meiser Andrea, Mikus Maria, Monk Philip, N. Mores, Naz Shama, K. Nething, Nicholas Ben, U. Nihlén, Nilsson Peter, R. Niven, B. Nordlund, S. Nsubuga, Pacino Antonio, Palkonen Susanna, L. Pahus, J. Pellet, Pennazza Giorgio, Petrén Anne, Pink Sandy, C. Pison, Rahman‐Amin Malayka, Ravanetti Lara, Ray Emma, Reinke Stacey, Reynolds Leanne, K. Riemann, Robberechts Martine, J. P. Rocha, C. Rossios, Russell Kirsty, Rutgers Michael, G. Santini, Santonico Marco, M. Saqi, Schoelch Corinna, S. Scott, N. Sehgal, A. Selby, Sjödin Marcus, Smids Barbara, Smith Caroline, Smith Jessica, M. Smith Katherine, P. Söderman, A. Sogbesan, F. Spycher, Staykova Doroteya, S. Stephan, J. Stokholm, K. Strandberg, M. Sunther, M. Szentkereszty, L. Tamasi, K. Tariq, Thörngren John‐Olof, Thorsen Jonathan, S. Valente, W. M. van Aalderen, Marianne van de Pol, C. M. van Drunen, Van Eyll Jonathan, Versnel Jenny, Vink Anton, C. von Garnier, A. Vyas, A. H. Wagener, Wald Frans, Walker Samantha, Ward Jonathan, Wetzel Kristiane, Wiegman Coen, Z. Weiszhart, Williams Siân, Yang Xian, Yeyasingham Elizabeth, W. Yu, W. Zetterquist, Z. Zolkipli, A. H. Zwinderman, Lega Italiano Anti Fumo, A. B. Aerocrine, N. V. Amgen, Merck Sharp, Martina Gahlemann, Luigi Visintin, Hazel Evans, Martine Puhl, Lina Buzermaniene, Val Hudson, Laura Bond, Pim de Boer, Guy Widdershoven, Ralf Sigmund, David Supple, Dominique Hamerlijnck, Jenny Negus, Juliёtte Kamphuis

**Affiliations:** ^1^ National Heart & Lung Institute, Imperial College London London UK; ^2^ Royal Brompton Hospital, Guy's and St Thomas' NHS Foundation Trust London UK; ^3^ Institute of Health and Environment, Seoul National University Seoul Republic of Korea; ^4^ Sysmex R&D Center Europe GmbH Hamburg Germany; ^5^ Data Science Institute, Imperial College London London UK; ^6^ Department of Public Health Sciences Graduate School of Public Health, Seoul National University Seoul Republic of Korea; ^7^ Department of Allergy and Clinical Immunology Asan Medical Center, University of Ulsan College of Medicine Seoul Korea

**Keywords:** biologic, CXCL9, duration, eosinophils, periostin

## Abstract

**Background:**

Longer asthma duration predicts non‐remission in Type‐2 (T2) biologic‐treated severe asthma, but underlying mechanisms remain unclear. We investigated associations between asthma duration and inflammatory biomarkers that may explain differential biologic response.

**Methods:**

We analysed cross‐sectional data from adults with severe asthma in U‐BIOPRED. Asthma duration was defined as years from diagnosis to study entry. T2 and non‐T2 biomarkers were measured in blood and sputum. Identical assays were performed in PRISM, a validation cohort of biologic‐initiators. Multivariable regression models adjusted for age, sex, ethnicity, BMI, smoking and oral corticosteroid dose. Associations between duration‐related biomarkers and 12‐month biologic remission were explored in PRISM.

**Results:**

In U‐BIOPRED (*n* = 411), median asthma duration was 23 years (IQR 12–38). Longer duration was associated with higher non‐T2 biomarkers including sputum CXCL9 (*β* = 0.024, 95% CI 0.011–0.038), sputum IL‐6 (*β* = 0.024, 95% CI 0.011–0.037) and sputum neutrophils (*β* = 0.009, 95% CI 0.001–0.017), but lower T2 biomarkers including plasma periostin (*β* = −0.006, 95% CI −0.009 to −0.003), eosinophils (blood: *β* = −0.002, 95% CI −0.003 to −0.001; sputum: *β* = −0.023, 95% CI −0.035 to −0.011), sputum EDN (*β* = −0.032, 95% CI −0.049 to −0.014) and FeNO (*β* = −0.006, 95% CI −0.010 to −0.001). PRISM (*n* = 474) confirmed these associations. Higher sputum CXCL9 was associated with reduced remission in anti‐IL‐4Rα‐treated patients with asthma duration ≥ 20 years (*β* = −1.73, 95% CI −3.180 to −0.276).

**Conclusion:**

Longer asthma duration was associated with elevated airway non‐T2 inflammation and reduced systemic and airway T2 inflammation. This highlights the importance of airway biomarker sampling and suggests combined T2 and non‐T2 therapeutic strategies may be needed to achieve remission in long‐standing asthma.

**Trial Registration:**

ClinicalTrials.gov identifier: NCT05164939

## Graphical abstract

Longer asthma duration is associated with increased airway non‐T2 inflammation and reduced airway and systemic T2 inflammation. Sputum CXCL9 is independently associated with duration and may predict a reduced poor response to anti‐IL‐4Rα therapy. Airway sampling and combined T2 and non‐T2 targeted strategies may be needed in long‐standing asthma. CXCL9, C‐X‐C motif chemokine ligand 9; EDN, eosinophil‐derived neurotoxin; FeNO, fractional exhaled nitric oxide; IL‐6, interleukin 6; PRISM, Precision Medicine Intervention in Severe Asthma; T2, type 2; U‐BIOPRED, Unbiased Biomarkers for the Prediction of Respiratory Disease Outcomes.

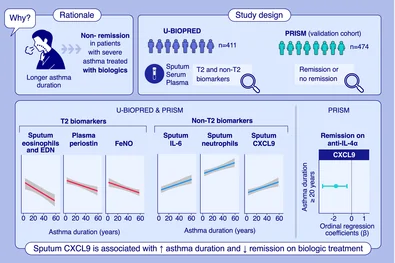

**Abbreviations**
CXCL9: C‐X‐C Motif Chemokine Ligand 9
EDN: eosinophil‐derived neurotoxin
FeNO: fractional exhaled nitric oxide
IL: interleukin
PRISM: Precision Medicine Intervention in Severe Asthma (Korea–UK biologic study)
U‐BIOPRED: Unbiased Biomarkers for the Prediction of Respiratory Disease Outcomes

## 1 Introduction

Severe asthma is a chronic respiratory disease characterised by variable inflammation and frequent exacerbations [1]. The most common severe asthma endotype is type 2 (T2) inflammation. Monoclonal antibody therapies (biologics) targeting T2 pathways have proven highly effective at reducing exacerbations [2].

The primary biologic treatment goal is to achieve clinical remission, commonly defined as no exacerbations or oral corticosteroid requirements, complete symptom control and normalisation of lung function. However, remission is achieved in only 30%–38% of severe asthma patients receiving biologic treatment. Longer asthma duration has been consistently identified as an independent predictor of non‐remission [3]. This association persists even in patients with T2‐high endotypes who would be expected to respond to biologic therapy [3]. The mechanisms underlying the relationship between asthma duration and remission on biologic treatments remain unclear.

Most studies identifying the relationship between asthma duration and reduced remission on biologics are based on clinical data with limited inflammatory profiling [3, 4, 5]. Biomarker assessments in these studies are typically restricted to conventional measures such as blood eosinophil count and fractional exhaled nitric oxide (FeNO).

The Unbiased Biomarkers for the Prediction of Respiratory Disease Outcomes (U‐BIOPRED) study recruited patients with severe asthma and performed comprehensive inflammatory profiling using blood and induced sputum [6]. This dataset provides a unique opportunity to characterise inflammatory profiles across the spectrum of asthma duration and identify biomarkers associated with longer disease duration.

Understanding how asthma duration relates to inflammatory profile may explain differential responses to T2‐targeted biologics. Therefore, we aimed to: (1) identify biomarkers associated with longer asthma duration and (2) explore whether these biomarkers are associated with non‐remission in severe asthma patients receiving biologic therapy.

## 2 Methods

### 2.1 Study Design and Population

We conducted a cross‐sectional analysis of baseline clinical, blood and sputum data from U‐BIOPRED [6]. Participants were recruited from 11 specialist asthma centres across Europe. We included participants aged ≥ 18 years with severe asthma meeting ATS/ERS criteria, defined as uncontrolled asthma despite high‐dose inhaled corticosteroids plus a second controller and/or systemic corticosteroids [1], with historical evidence of airflow reversibility, variability, or airway hyperresponsiveness confirming asthma diagnosis. Biomarkers were measured at baseline and 1‐year follow‐up visits [6].

The validation cohort consisted of patients with severe asthma who met the same ATS/ERS criteria and were drawn from the Precision Medicine Intervention in Severe Asthma (PRISM) study, where full eligibility criteria, including ATS/ERS severe asthma, are described by Lee et al. [7]. PRISM is a prospective observational study of biologic‐naïve patients recruited in Korea and the UK who were initiating biologic treatment. Patients were followed up for 12 months to evaluate treatment outcomes, with baseline biomarker assessments performed prior to treatment initiation [7].

Asthma duration was calculated as the difference between age at asthma diagnosis and age at baseline visit. Participants with missing asthma duration data were excluded.

U‐BIOPRED was approved by ethics committees at all participating institutions and registered on ClinicalTrials.gov (NCT01982162). PRISM‐Korea was approved by the Institutional Review Board of participating centres (IRAS: 2019–1676), PRISM‐UK study was approved by the UK Health and Care Research Wales (REC 25/WA/0146, IRAS: 354503) and registered on ClinicalTrials.gov (NCT05164939). All participants provided written informed consent.

### 2.2 Outcomes and Covariates

Standard clinical biomarkers included blood eosinophil and neutrophil counts, total Immunoglobin E (IgE), C‐reactive protein (CRP), fractional exhaled nitric oxide (FeNO) and induced sputum granulocyte percentages. Methods for obtaining and processing induced sputum and reporting granulocyte percentages have been reported previously [8, 9].

In U‐BIOPRED, 18 sputum and 24 plasma biomarkers were measured. In PRISM, 10 sputum, 6 plasma and 16 serum biomarkers were measured. Induced sputum and blood cytokines and mediators were measured using a fully automated immunoassay analyser (HISCL‐5000 or HISCL‐800; Sysmex, Hyogo, Japan). Interleukin‐17 (IL‐17) levels were measured using a fully automated highly sensitive immunoassay analyser (HI‐1000; Sysmex).

In U‐BIOPRED, to maximise biomarker availability, measurements from the 1‐year follow‐up visit were substituted when baseline samples were unavailable. This substitution was required for 19% of samples (Table S1). To assess potential selection bias introduced by missing sputum data, we compared baseline demographic and clinical characteristics between patients with and without available sputum biomarker measurements.

In PRISM, clinical remission at 12 months after biologic initiation was assessed using an ordinal score ranging from 0 to 4 based on the number of remission criteria met: (1) no oral corticosteroid use for exacerbations, (2) maintenance oral corticosteroid dose reduced to ≤ 5 mg, (3) complete symptom control (ACQ < 1.5 in the UK; ACT≥ 20 in Korea) and (4) normalisation (percent predicted ≥ 80%) or ≥ 10% improvement in FEV_1_ percent predicted from baseline.

Covariates included patient demographics, smoking history, body mass index (BMI), asthma medication history (including maintenance oral corticosteroid use and dose) and spirometry. Daily prednisolone‐equivalent doses were calculated for patients receiving alternative glucocorticoids.

### 2.3 Statistical Methods

Descriptive group comparisons used unpaired *t*‐tests and one‐way ANOVA for parametric continuous variables, Wilcoxon‐Mann–Whitney and Kruskal–Wallis tests for non‐parametric continuous variables and chi‐squared tests for categorical variables.

Baseline associations between biomarker concentrations and asthma duration were assessed separately for each analyte. Most biomarker measurements demonstrated right‐skewed distributions, often with a mass at zero. Biomarker levels were therefore log‐transformed (log[*x* + 1]) to improve normality while handling zero values.

Linear regression tested associations between asthma duration and log‐transformed biomarker levels. Where standard log‐transformation was insufficient to normalise the data, typically where many participants had zero values alongside a continuous range of positive measurements, Tweedie generalised linear modelling was applied as it is better suited to handling this type of data. All multivariable models were adjusted for age, sex, ethnicity, BMI, smoking status (never/former/current) and oral corticosteroid dose (prednisolone equivalent mg/day).

Due to population heterogeneity in the PRISM cohort, models were fitted separately for Korean and UK participants, and population‐specific estimates were subsequently combined using inverse‐variance weighted meta‐analysis, with standard error used as the within‐population variance.

In the discovery analysis using U‐BIOPRED data, statistical significance was defined as adjusted *p*‐value < 0.05 after Benjamini‐Hochberg correction. In the validation analysis using PRISM baseline data, a *p*‐value of *p* < 0.05 was used to define significance for pre‐specified biomarkers, while additional biomarkers were evaluated in an exploratory framework with adjustment for multiple testing.

Sensitivity analyses were performed in U‐BIOPRED and PRISM to assess the robustness of primary findings: (1) regression models were repeated after stratifying by age of asthma onset into childhood‐onset (< 18 years, *n* = 80) and adult‐onset (≥ 18 years, *n* = 150) subgroups, (2) variance inflation factors (VIF) were calculated for all covariates to assess potential collinearity between asthma duration and age, with regression models additionally repeated after stratifying by median age, and (3) in U‐BIOPRED only, all primary models were repeated excluding participants receiving omalizumab at baseline, to assess whether anti‐IgE therapy confounded the associations between asthma duration and inflammatory biomarkers.

Exploratory analysis evaluating associations between baseline biomarkers and clinical remission were conducted in the PRISM cohort among patients treated with anti‐IL‐4Rα or anti‐IL‐5/5Rα biologics. Associations were assessed using cumulative logistic regression models that includes an interaction term between asthma duration and log‐transformed biomarker levels, allowing the biomarker‐remission relationship to vary according to asthma duration. Models were adjusted for the aforementioned confounders, and Hessian‐based standard errors were used for inference. Patients were stratified by biologic class (anti‐IL‐4Rα and anti‐IL‐5/5Rα) and asthma duration (< 20 and ≥ 20 years since onset). All analyses were performed using R versions 4.3.2 or 4.1.1 for U‐BIOPRED and PRISM analyses, respectively.

## 3 Results

### 3.1 Study Populations

Of 422 severe asthma patients in U‐BIOPRED, 411 (97%) had data for age of asthma onset and were included in the analysis. The median asthma duration was 23 years (Q1, Q3: 12, 38). Median age was 53 years (Q1, Q3: 44, 62), and 62% were female. Forty‐two patients (10%) were current smokers, 113 (27%) were ex‐smokers and 256 (62%) were never smokers. Half of patients (51%, *n* = 210) were receiving daily oral corticosteroids (Table 1).

**TABLE 1:** Study population characteristics of U‐BIOPRED and PRISM.

	U‐BIOPRED (*n* = 421)	PRISM (*n* = 476)
Age (years)	54.0 [44.0, 62.0]	54.5 [46.0, 64.0]
Age at asthma diagnosis (years)	26.0 [9.00, 42.0]	42.0 [31.2, 51.0]
(missing)	10 (2.4%)	2 (0.4%)
Asthma duration (years)	23.0 [12.0, 37.5]	10.0 [5.0, 20.0]
(missing)	10 (2.4%)	2 (0.4%)
Female	261 (62.0%)	268 (56%)
Smoking status		
Never	264 (62.7%)	252 (53%)
Ex‐smoker	115 (27.3%)	183 (39%)
Current	42 (10.0%)	39 (8.2%)
(missing)	0	2 (0.4%)
BMI, kg/m^2^	28.1 [24.6, 33.3]	24.8 [22.3, 28.3]
(missing)	0	1 (0.2%)
Daily OCS dose, mg	0 [0, 12.5]	0.0 [0.0, 4.6]
FeNO	26.0 [15.0, 47.0]	52.0 [29.0, 89.0]
(missing)	27 (6.4%)	16 (3.4%)
FEV1 (% predicted)	66.8 [51.2, 83.5]	66.0 [52.0, 79.0]
(missing)	3 (0.7%)	3 (0.6%)
FEV1/FVC (%)	63.8 [53.1, 73.8]	65.0 [57.0, 73.2]
(missing)	3 (0.7%)	4 (0.8%)
Blood eosinophil counts, cells/μL	0.200 [0.100, 0.400]	0.500 [0.200, 0.800]
(missing)	13 (3.1%)	3 (0.6%)
Sputum eosinophil (%)	2.9 [0.4, 16.5]	5.0 [0.0, 29.0]
(missing)	240 (57.0%)	140 (29.4%)
Sputum neutrophil (%)	54.0 [34.2, 74.4]	59.0 [20.0, 82.0]
(missing)	240 (57.0%)	167 (35.1%)
Total IgE, IU/mL	120 [47.0, 348]	227.5 [84.2, 547.0]
(missing)	10 (2.4%)	70 (14.7%)
CRP, mg/dL	2.20 [0.930, 4.80]	0.2 [0.1, 0.6]
(missing)	8 (1.9%)	126 (26.5%)

*Note:* Results are shown as *n* (%) or median [Q1, Q3].

### 3.2 Cytokines and Mediators

Biomarker measurements were available for 237 patients (58%). Demographics and clinical characteristics were similar between patients with and without (*n* = 184) available sputum measurements, except for FEV1/FVC, which was lower in those with available sputum results (Table S2).

Univariable analysis showed that asthma duration was positively associated with sputum CXCL9 (*β* = 2.30, 95% CI 1.02–3.58), IL‐6 (*β* = 2.16, 95% CI 0.88–3.43) and CCL3 (*β* = 1.15, 95% CI 0.35–1.95), but negatively associated with sputum EDN (*β* = −1.26, 95% CI −2.21, −0.30) (Table S3).

**FIGURE 1:**
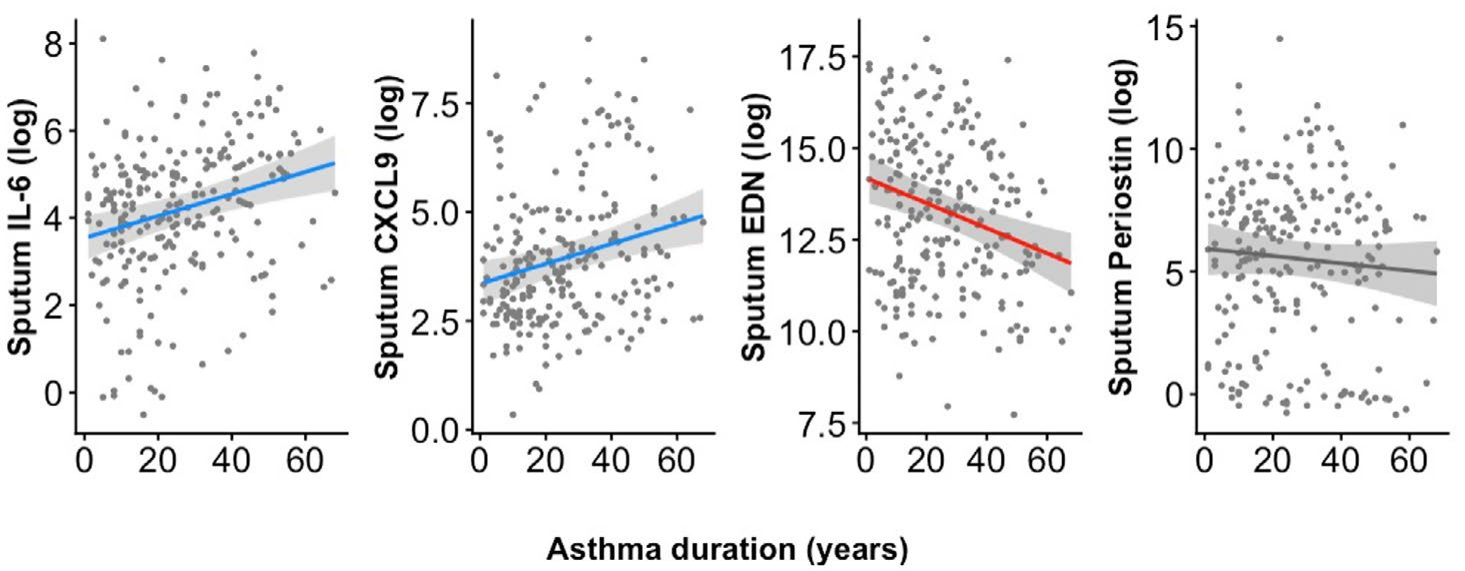
Multivariable linear regression analyses examining the relationship between asthma duration and biomarker levels in *induced sputum*. Models were adjusted for age, sex, race, smoking status, body mass index and daily prednisolone dose. Regression lines are colour‐coded by inflammatory phenotype and statistical significance: red lines indicate significant associations with T2 biomarkers (sputum EDN); blue lines indicate significant associations with non‐T2 biomarkers (sputum IL‐6, sputum CXCL9); grey lines indicate non‐significant associations. Longer asthma duration was associated with lower levels of T2 biomarkers and higher levels of non‐T2 biomarkers in sputum.

In multivariable analysis adjusted for age, sex, ethnicity, BMI, smoking status and oral corticosteroid dose, the associations between asthma duration and sputum CXCL9 (*β* = 0.02, 95% CI 0.01–0.04), IL‐6 (*β* = 0.02, 95% CI 0.01–0.04) and EDN (*β* = −0.03, 95% CI −0.05, −0.01) remained significant, indicating that longer asthma duration was associated with elevated non‐T2 biomarkers and reduced T2 biomarkers in induced sputum (Figure 1, Table 2, Table S4).

**TABLE 2:** Multivariable linear regression analysis of the association between asthma duration and airway and systemic inflammatory biomarkers in the U‐BIOPRED cohort.

Biomarker	*n*	Coefficient	5% CI	95% CI	*p*
Sputum CXCL9	231	**0.024**	**0.011**	**0.038**	**0.001**
Sputum EDN	231	**−0.032**	**−0.049**	**−0.014**	**< 0.001**
Sputum IL‐6	230	**0.024**	**0.010**	**0.037**	**0.001**
Sputum periostin total	229	−0.015	−0.044	0.013	0.297
Plasma CXCL9	231	−0.001	−0.007	0.006	0.866
Plasma EDN	229	−0.005	−0.010	−0.0001	0.047
Plasma IL‐6	231	−0.004	−0.010	0.002	0.227
Plasma periostin total	229	**−0.006**	**−0.009**	**−0.003**	**< 0.001**
Blood eosinophil count (×10^3^ μL)	398	**−0.002**	**−0.003**	**−0.001**	**0.003**
Total IgE (IU/mL)	402	−0.003	−0.012	0.007	0.565
Sputum eosinophil percentage (%)	180	**−0.023**	**−0.035**	**−0.011**	**< 0.001**
Sputum neutrophil percentage (%)	180	**0.009**	**0.001**	**0.017**	**0.026**
FeNO (ppb)	385	**−0.006**	**−0.010**	**−0.001**	**0.022**

*Note:* Statistically significant results are highlighted in bold.

**FIGURE 2:**
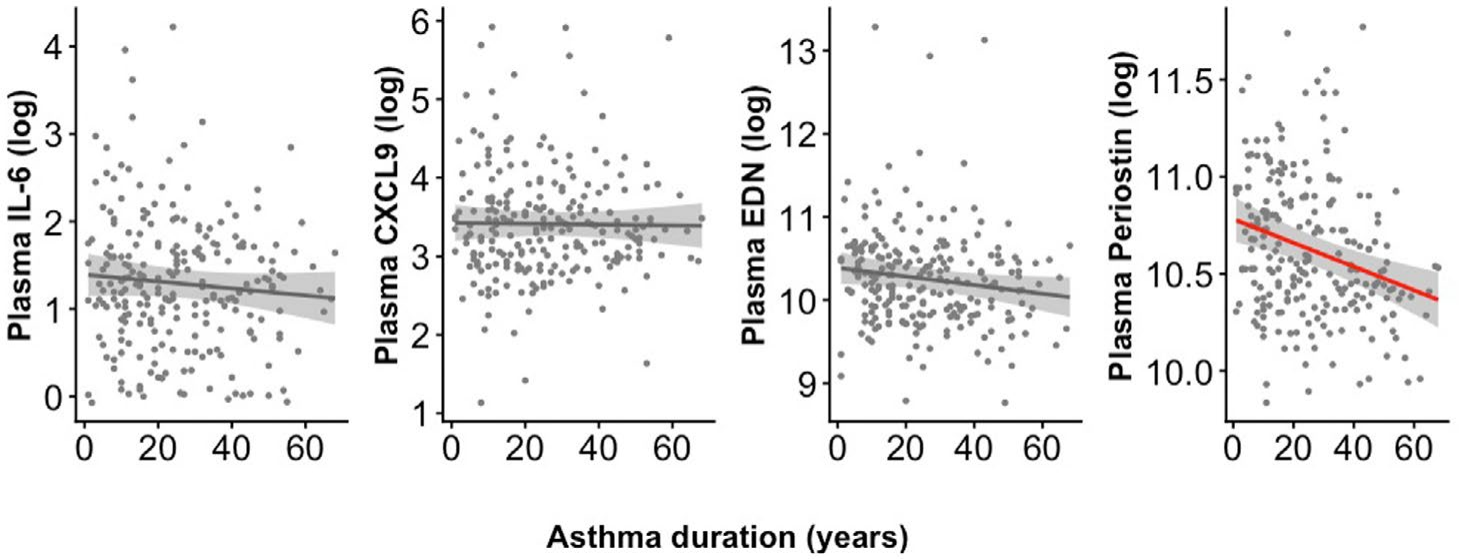
Multivariable linear regression analyses examining the relationship between asthma duration and biomarker levels in *plasma*. Models were adjusted for age, sex, race, smoking status, body mass index and daily prednisolone dose. Regression lines are colour‐coded by inflammatory phenotype and statistical significance: red lines indicate significant associations with T2 biomarkers (plasma periostin); grey lines indicate non‐significant associations. Longer asthma duration was associated with lower levels of T2 biomarker in plasma.

In plasma, univariable analysis showed no significant associations between asthma duration and any biomarkers, including CXCL9 (*β* = 1.18, 95% CI −0.86, 4.49), IL‐6 (*β* = −1.07, 95% CI −3.83, 1.70), CCL3 (*β* = 0.84, 95% CI −3.58, 5.25), or EDN (*β* = −1.04, 95% CI −4.43, 2.35). Similarly, multivariable analysis showed no significant associations for these markers. However, after adjusting for confounders in the multivariable model, plasma periostin, a T2 biomarker, demonstrated a significant negative association with asthma duration (*β* = −0.01, 95% CI −0.01 to −0.003) (Figure 2, Table 2, Tables S5 and S6).

### 3.3 Current Biomarkers

**FIGURE 3:**
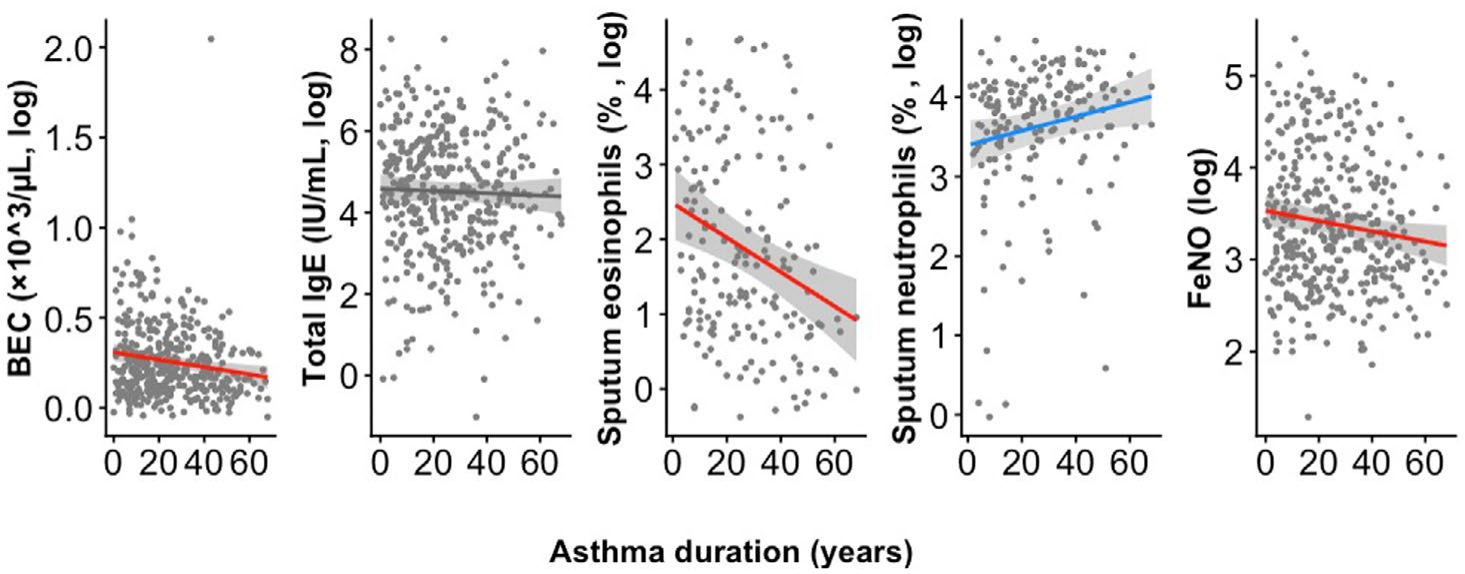
Multivariable linear regression analyses examining the relationship between asthma duration and current clinical biomarkers. Models were adjusted for age, sex, race, smoking status, body mass index and daily prednisolone dose. Regression lines are colour‐coded by inflammatory phenotype and statistical significance: red lines indicate significant associations with T2 biomarkers (blood and sputum eosinophils, FeNO); blue lines indicate significant associations with non‐T2 biomarker (sputum neutrophils); grey lines indicate non‐significant associations. Longer asthma duration was associated with lower levels of T2 biomarkers and higher levels of non‐T2 biomarkers.

In univariable analysis, asthma duration was negatively associated with sputum eosinophils (*β* = −2.54, 95% CI −4.27 to −0.80). This association remained significant in multivariable analysis (*β* = −0.02, 95% CI −0.04 to −0.01) (Table S7). Additionally, sputum neutrophils (*β* = 0.009, 95% CI 0.001–0.017), FeNO (*β* = −0.006, 95% CI −0.010 to −0.001) and blood eosinophils (*β* = −0.002, 95% CI −0.003 to −0.001) became significantly associated with asthma duration after adjusting for demographics, smoking history and oral corticosteroid dose. Consistent with cytokine/chemokine findings, longer asthma duration was associated with higher non‐T2 biomarkers (sputum neutrophils) and lower T2 biomarkers (sputum eosinophils, blood eosinophils and FeNO) (Figure 3, Table 2, Table S8).

### 3.4 Validation

The PRISM cohort comprised 474 severe asthma patients initiating biologic therapy. The median asthma duration was substantially shorter than in U‐BIOPRED (Median 10 years, Q1, Q3: 5, 20). Age and smoking status distribution were similar to U‐BIOPRED. However, the proportion of females was lower (55% vs. 62%), as was median BMI (24 vs. 28 kg/m^2^) and the proportion receiving oral corticosteroids (33% vs. 51%). Patients had higher FeNO and blood and sputum eosinophil counts compared to the U‐BIOPRED cohort, consistent with enrichment for biologic‐eligible patients (Table 1). The difference in clinical characteristics between the PRISM UK and Korean populations is shown in Table S9.

In the PRISM cohort, multivariable analysis validated key findings from U‐BIOPRED. Asthma duration was positively associated with sputum CXCL9 (*β* = 0.042, 95% CI 0.019–0.065) and negatively associated with plasma periostin (*β* = −0.004, 95% CI −0.008 to −0.0003). When considering all assessed biomarkers, additional associations were identified after adjustment for multiple testing. Sputum CCL17 and sputum TNF‐α were positively associated with asthma duration (*β* = 0.031, 95% CI 0.012–0.049; *β* = 0.015, 95% CI 0.004–0.025, respectively), while serum IL‐16 showed a negative association (*β* = −0.010, 95% CI −0.016 to −0.003) (Table S10).

Multivariable analysis in PRISM also confirmed the associations between asthma duration and current clinical biomarkers observed in U‐BIOPRED. Asthma duration was positively associated with sputum neutrophils (*β* = 0.40, 95% CI 0.071–0.730) and negatively associated with sputum eosinophils (*β* = −0.27, 95% CI −0.513 to −0.025), blood eosinophils (*β* = −6.43, 95% CI −11.207 to −1.662) and FeNO (*β* = −0.82, 95% CI −1.173 to −0.471) (Table S11).

### 3.5 Biologic Response

In PRISM, 257 patients received anti‐IL‐4Rα (dupilumab), 127 received anti‐IL‐5 (mepolizumab) and 78 received anti‐IL‐5Rα (benralizumab). Baseline sputum CXCL9 measurements were available for 184 (72%), 65 (51%) and 36 (46%) patients, respectively.

Among patients who completed 12 months of treatment with available remission outcomes and baseline sputum CXCL9 (*n* = 214), clinical remission was achieved in 67/142 (47%) of anti‐IL‐4Rα‐treated patients, 20/44 (45%) of anti‐IL‐5‐treated patients and 17/28 (61%) of anti‐IL‐5Rα‐treated patients (Table S12).

**FIGURE 4:**
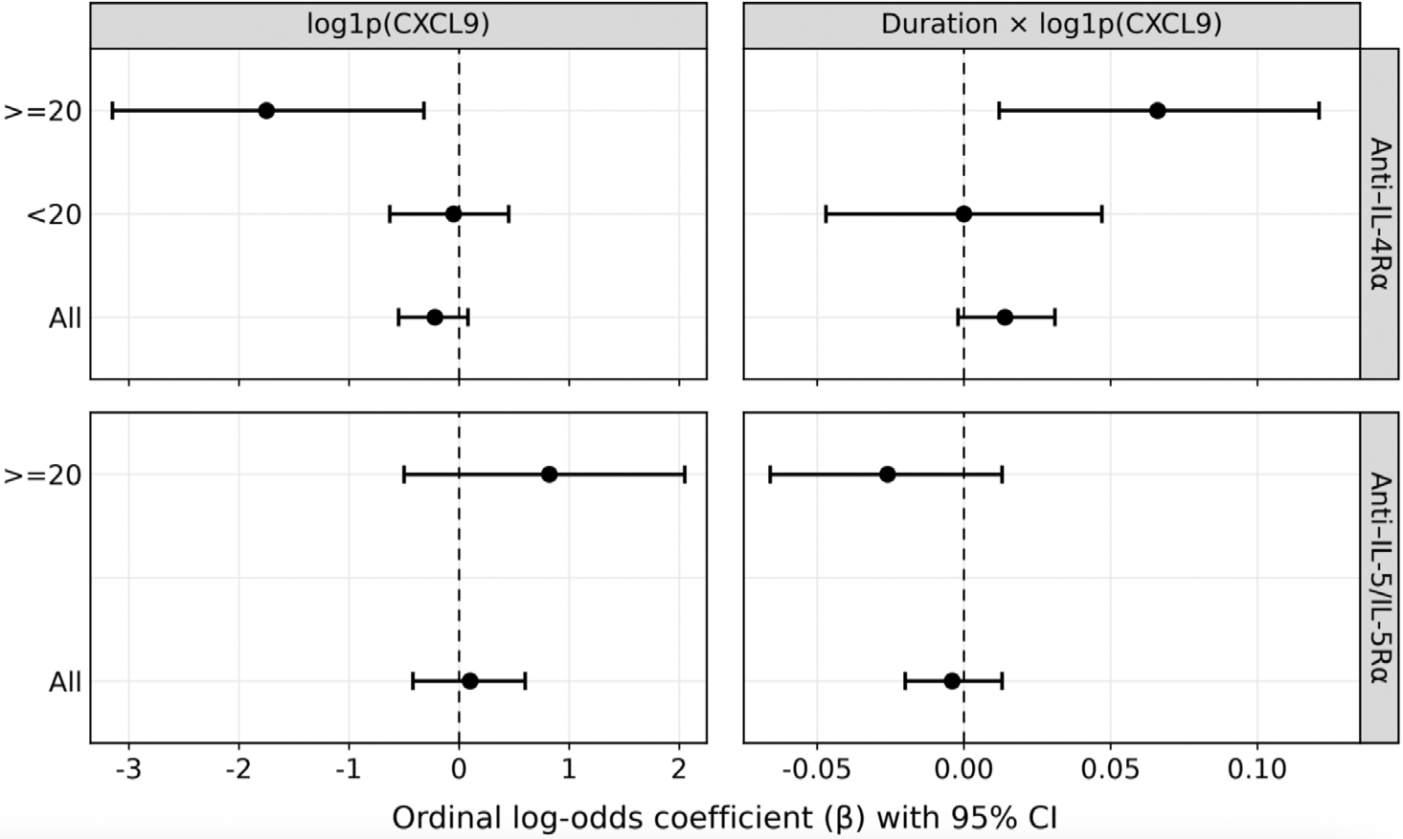
Association between sputum CXCL9 and remission score in patients treated with anti‐IL‐4Rα and anti‐IL‐5/IL‐5Rα therapies. Forest plots show ordinal regression coefficients (*β*) with 95% CIs for log‐transformed sputum CXCL9 and the Duration × CXCL9 interaction term, across all patients and stratified by asthma duration (< 20 and ≥ 20 years). In anti‐IL‐4Rα‐treated patients, sputum CXCL9 was negatively associated with remission in those with duration ≥ 20 years (*β* = −1.73, *p* = 0.020), with a significant duration–CXCL9 interaction (*β* = 0.07, *p* = 0.017); no association was observed in those with duration < 20 years. Sputum CXCL9 was not associated with remission in anti‐IL‐5/IL‐5Rα‐treated patients with duration ≥ 20 years; the < 20 years subgroup could not be analysed due to model non‐convergence from sparse data.

Multivariable modelling demonstrated that baseline sputum CXCL9 was not associated with remission score in the overall cohort receiving anti‐IL‐4Rα or anti‐IL‐5/anti‐IL‐5Rα (Table S13). However, when stratified by asthma duration, sputum CXCL9 was negatively associated with remission in anti‐IL‐4Rα treated patients with disease duration ≥ 20 years (*β* = −1.73, 95% CI −3.180 to −0.276). The interaction term between asthma duration and CXCL9 was significant, indicating that the negative association between CXCL9 and remission weakened as asthma duration increased beyond 20 years (Figure 4, Table S14).

In contrast, no significant association between sputum CXCL9 and remission was observed in the anti‐IL‐5/IL‐5Rα‐treated group, either overall or when stratified by asthma duration. The interaction term between asthma duration and CXCL9 was also non‐significant.

### 3.6 Sensitivity Analyses

Duration‐associated inflammatory trends were directionally consistent with the primary analysis across onset subgroups in both cohorts, despite longer median asthma duration in the childhood‐onset group (40 years [IQR 25–48] vs. 16 years [IQR 8–25] in U‐BIOPRED). In U‐BIOPRED, sputum CXCL9 (positive), EDN (negative) and plasma periostin (negative) all maintained consistent coefficient directions in both subgroups (Tables S15 and S16). In PRISM, sputum CXCL9 associations were significant in the adult‐onset group (*β* = 0.062, 95% CI 0.030–0.094) and directionally consistent in the childhood‐onset group (*β* = 0.076, 95% CI −0.009 to 0.162), with plasma periostin similarly consistent across both subgroups (Table S17).

VIF values for duration and age were consistently between 1 and 2 across all sputum biomarker models, indicating negligible multicollinearity (Tables S18–S20). Age‐stratified analyses confirmed that duration‐associated inflammatory trends were preserved in both younger and older subgroups (Tables S21 and S22).

In a sensitivity analysis restricted to omalizumab‐naïve participants (*n* = 358), all primary associations were preserved and an additional non‐T2 sputum biomarker (CCL3) reaching statistical significance, further supporting the primary findings (Table S23).

## 4 Discussion

In this study, we demonstrated that longer asthma duration is associated with elevated non‐T2 biomarkers in sputum and reduced T2 biomarkers in both sputum and blood. Specifically, longer disease duration was associated with higher sputum CXCL9 and neutrophils, alongside lower sputum EDN, FeNO, plasma periostin and eosinophils in both blood and sputum. These findings were consistent across two independent severe asthma cohorts using identical assays. Critically, we identified that elevated sputum CXCL9, a type 1 interferon‐induced chemokine, was associated with reduced remission in anti‐IL‐4Rα‐treated patients with disease duration ≥ 20 years. Sputum CXCL9 may serve as a pragmatic airway biomarker to identify patients without long‐standing asthma who are unlikely to achieve remission with T2‐targeted biologics alone. Collectively, these findings help understand why patients with longer disease duration are less likely to achieve clinical remission despite meeting conventional eligibility criteria for biologic therapy.

Several longitudinal asthma cohorts have now been followed for multiple decades, providing valuable insights into disease trajectories across the lifespan. The Tasmanian Longitudinal Health Study (TAHS) has tracked participants for over 46 years [10], while the Saguenay–Lac‐Saint‐Jean Asthma Family Cohort followed 125 participants over 10–20 years [11]. Other notable cohorts include a Korean study with 10‐year follow‐up of severe and non‐severe asthma patients [12], the Copenhagen General Population Study with 10‐year follow‐up examining T2 inflammation and lung function decline [13], and the Glenfield Asthma Cohort assessing airway inflammatory patterns over 6 years [14]. However, these longitudinal studies are limited in biomarker assessments over time, with some measuring blood eosinophils and FeNO longitudinally and others measuring sputum granulocytes over shorter periods. No studies have comprehensively sampled biomarker profiles across the entire lifespan of asthma patients. Therefore, cross‐sectional data with comprehensive inflammatory profiling currently offers the best approach to understanding different inflammatory biomarker phenotypes in patients with varying asthma durations.

A key strength of our study is the U‐BIOPRED design, which specifically aimed to endotype severe asthma through robust clinical data collection and detailed phenotyping. We analysed a broad biomarker panel incorporating matched blood and sputum samples to assess different inflammatory compartments. Importantly, the positive association between asthma duration and non‐T2 markers (CXCL9, neutrophils) was detected only in sputum, not in blood. This compartment‐specific finding suggests that sputum may be superior to blood for detecting non‐T2 inflammation in chronic asthma, indicating that non‐T2 inflammation in longer‐duration disease predominantly localises to the airways rather than systemically. In contrast, the negative association between asthma duration and T2 biomarkers was observed across both airway (FeNO and sputum) and systemic (blood) compartments. These findings are consistent with previous observations that blood and airway biomarkers do not always correlate, reflecting compartment‐specific differences in airway inflammation [15].

This airway‐specific accumulation of non‐T2 inflammation in long‐standing asthma may reflect several converging factors, including changes in airway microbiota, repeated exposure to corticosteroids and antibiotics, and the natural inflammatory trajectory towards a less T2‐dominant asthma phenotype over time. Collectively, these processes may lead to a residual inflammatory burden that T2‐targeted biologics alone cannot adequately address. Indeed, chronic bacterial colonisation of the lower airways is common in severe asthma and strongly associated with longer disease duration. Studies have demonstrated bacterial colonisation with *Haemophilus influenzae* , *Moraxella catarrhalis* and *Streptococcus pneumoniae* is associated with airway neutrophilic inflammation and corticosteroid resistance [16, 17, 18]. Versi et al. showed that severe asthma patients with high relative abundance of *Haemophilus influenzae* had a median asthma duration of 38 years [19]. Similarly, Kuo et al. identified a transcriptomic cluster characterised by elevated sputum neutrophilia, interferon, TNF‐α and inflammasome‐associated genes. These patients had significantly longer asthma duration compared to the T2‐high patients (median 32 vs. 21 years) [20]. Collectively, these findings suggest that duration‐dependent changes in airway microbiota may be a factor in sputum neutrophilia and subsequent reduced biologic response seen in chronic asthma.

We identified sputum CXCL9 as independently associated with longer asthma duration, and critically, elevated CXCL9 predicted reduced remission on dupilumab in patients with disease duration over 20 years. CXCL9, also known as monokine induced by interferon‐γ (MIG), is an interferon‐γ‐inducible chemokine that signals through CXCR3, alongside CXCL10 (IP‐10) and CXCL11 [21]. This chemokine recruits Th1 cells and neutrophils to inflamed tissues via CXCR3, contributing to tissue damage and reduced lung function [22, 23]. Importantly, Th1 inflammation driven by CXCL9 and related chemokines has been linked with corticosteroid resistance in severe asthma. Gauthier et al. demonstrated that airway CXCL10 expression reflects resistance to corticosteroid treatment, with corticosteroids paradoxically enhancing T1 inflammation in some patients [23].

Our findings extend previous observations linking elevated CXCL9 to older age [24] by demonstrating that its association with asthma duration is independent of age. This suggests that disease chronicity, rather than ageing per se, drives this inflammatory shift towards Th1‐mediated pathology. Additionally, CXCL9 antagonises CCR3‐mediated eosinophil recruitment [25], potentially reducing the efficacy of T2‐targeted treatments in prolonged asthma who develop concurrent Th1 inflammation.

Emerging evidence indicates that CXCL9 may serve as a marker of Th1 inflammation in the context of eosinophilic asthma [26]. Gauthier et al. reported that CXCL9 and CXCL10 mRNA expression closely correlated with CCL5 in severe asthma (SARP‐III), with elevated bronchoalveolar lavage fluid CCL5 levels specific to a T1‐high/T2‐variable lymphocytic phenotype [27]. These findings suggest that CCL5. May bridge Th1 and Th2 inflammatory pathways in severe asthma, raising the possibility that targeting this chemokine could address both inflammatory axes. Indeed, the CCR5 inhibitor maraviroc effectively blocked tissue‐resident memory T‐cell reactivation in Th1 inflammation in murine models [27], highlighting its translational potential. Future research should explore the role of CCL5 in prolonged asthma and evaluate its therapeutic potential, particularly in severe asthma with elevated T1 inflammation.

The positive association between asthma duration and sputum CCL17 in the PRISM cohort is particularly intriguing. CCL17 is predominantly a T2 biomarker that recruits CCR4+ Th2 cells and is typically elevated in allergic asthma [28, 29]. The PRISM cohort is enriched for dupilumab‐treated patients who were younger and with an allergic phenotype, which may explain the elevated CCL17. Studies have shown that elderly asthmatic patients have elevated CCL17 levels compared to younger patients [28], and CCL17 was positively correlated with total IgE and disease severity [30]. CCL17 may represent persistent allergic inflammation that coexists with emerging non‐T2 pathways in long‐standing disease. This may explain why we did not observe the same association in U‐BIOPRED, which had only 25% of patients with this allergic phenotype [31]. These findings further highlight the interplay between T2 and non‐T2 inflammatory pathways, reinforcing that long‐standing asthma is characterised by a complex inflammatory milieu rather than dominance by a single mediator.

Despite the advantages of a well‐characterised cohort with comprehensive biomarker profiling, several limitations should be acknowledged. First, the cross‐sectional design prevents assessment of longitudinal trends in individual patients, and all associations should be interpreted as correlative rather than mechanistic. Longitudinal studies with repeated biomarker sampling will be needed to confirm whether duration‐associated inflammatory changes represent a causal pathway to reduced biologic response. However, given that comprehensive biomarker profiling across the full spectrum of asthma duration has not been achieved in any existing longitudinal cohort, this cross‐sectional approach offers a valuable and currently irreplaceable insight into the biological characteristics of long‐standing asthma. Second, missing sputum biomarker data could introduce selection bias. Whilst key clinical characteristics were comparable between those with and without available sputum, patients with available sputum had lower FEV1/FVC ratios, suggesting this subgroup may have had greater airway remodelling. Third, bronchiectasis was not systematically recorded, and we cannot exclude the possibility that airway remodelling contributed to the non‐T2 airway signal in patients with longer disease duration. Fourth, asthma duration was calculated from patient‐reported age at diagnosis, introducing potential recall bias. Whilst minor inaccuracies are unlikely to materially alter our findings given the wide range of durations represented (1–68 years), a subset of patients may not recall childhood asthma or may have had undiagnosed early‐life symptoms, potentially leading to misclassification of disease duration. Fifth, given the considerable inter‐individual variability and modest individual effect sizes, findings should be interpreted at the pathway level rather than the individual biomarker level, and prospective validation in larger cohorts is required before clinical application. Finally, we acknowledge that asthma duration is an established predictor of non‐remission; characterising the underlying inflammatory mechanisms was therefore the primary objective, with biomarker‐remission associations treated as exploratory and requiring prospective validation.

In conclusion, airway sampling is essential for evaluating inflammatory profiles in long‐standing asthma. Sputum biomarkers, in particular CXCL9, may identify patients least likely to achieve remission with T2‐targeted biologics despite meeting conventional blood‐based eligibility criteria. The increased non‐T2 airway inflammation observed with longer disease duration suggests that T2‐targeting alone may be insufficient and combined therapeutic strategies addressing both T2 and non‐T2 inflammatory pathways warrant prospective investigation. To facilitate this in clinical practice, more accessible methods of airway sampling beyond sputum, such as nasosorption or breath volatiles, are needed. The role of airway infection and microbiome dysbiosis as drivers of non‐T2 airway inflammation in chronic asthma also warrants further investigation.

## Author Contributions

Study design: F.Y., S.J., T.‐B.K., S.W., I.M.A. and K.F.C. Analysis of U‐BIOPRED and PRISM: F.Y. and S.J. Analysis of biomarker samples: T.H. Drafted manuscript: F.Y. Interpretation of the analysis, revision, drafting, critical appraisal of the final draft: F.Y., S.S., T.H., C.I.B., N.Z.‐K., P.K.B., K.R., S.W., I.M.A., T.‐B.K. and K.F.C.

## Funding

U‐BIOPRED is funded by the Innovative Medicines Initiative Joint Undertaking (EU's Seventh Framework Programme and European Federation of Pharmaceutical Industries and Associations) and the eTRIKS project. PRISM‐Korea was supported by a grant from the Korea Health Technology R&D Project through the Korea Health Industry Development Institute (KHIDI), funded by the Ministry of Health & Welfare, Republic of Korea (grant number: RS‐2024‐00403700). PRISM‐UK was funded by a UKRI Medical Research Council grant MR/T01371/1.

## Ethics Statement

U‐BIOPRED was approved by ethics committees at all participating institutions and registered on ClinicalTrials.gov (NCT01982162). PRISM‐Korea was approved by the Institutional Review Board of participating centres (IRAS: 2019‐1676), PRISM‐UK study was approved by the UK Health and Care Research Wales (REC 25/WA/0146, IRAS: 354503).

## Consent

All participants provided written informed consent.

## Conflicts of Interest

F.Y. has received honoraria for attending meetings and speaker fees from AstraZeneca and GlaxoSmithKline, and is a member of the British Thoracic Society Asthma Advisory Group for work unrelated to this work; S.S. don't have any conflicts to declare in relation to this work; T.H. is an employee of the Sysmex corporation; C.I.B. has received research funding from the NIHR, Medical Research Council, European Respiratory Society, Asthma + Lung UK and AstraZeneca, outside the scope of this work; leadership roles in the British Thoracic Society and UK Primary Care Respiratory Society, N.K. has no conflicts to declare in relation to this work; P.K.B. has no conflicts to declare in relation to this work; K.R. has no conflicts to declare in relation to this work; I.M.A. has received investigator‐led awards from the EU‐IMI, MRC, EPSRC, GSK and Sanofi in addition to speaker fees and advisory board fees from AZ, GSK, Kineset, Sanofi and Sunovion for work unrelated to the current manuscript; S.W. has no conflicts to declare in relation to this work; T.‐B.K. has no conflicts to declare in relation to this work; K.F.C. is an Emeritus Senior Investigator of the UK National Institute for Health Research and has received honoraria for participation in advisory board meetings for GlaxoSmithKline, AstraZeneca, Novartis, Roche, Merck, Trevi, Nocion, Shionogi and Reckitt Benckiser, as well as for speaking engagements for GlaxoSmithKline, Novartis and AstraZeneca. He holds research grants from GlaxoSmithKline and Merck, which are paid to his institution.

## Supporting information


**Table S1:** U‐BIOPRED: Number of sputum results from baseline and follow‐up and total number included in study.
**Table S2:** U‐BIOPRED: study population characteristics of patients with cytokine/mediator biomarkers and those without.
**Table S3:** U‐BIOPRED Relationship between asthma duration (years) and *sputum* biomarkers—*univariable* linear regression with BH correction.
**Table S4:** U‐BIOPRED Relationship between asthma duration (years) and *sputum* biomarkers—*multivariable* linear regression with BH correction.
**Table S5:** U‐BIOPRED Relationship between asthma duration (years) and *plasma* biomarkers—*univariable* linear regression with BH correction.
**Table S6:** U‐BIOPRED Relationship between asthma duration (years) and *plasma* biomarkers—*multivariable* linear regression with BH correction.
**Table S7:** U‐BIOPRED Relationship between asthma duration (years) and *current* biomarkers—*univariable* linear regression with BH correction.
**Table S8:** U‐BIOPRED Relationship between asthma duration (years) and *current* biomarkers—*multivariable* linear regression with BH correction.
**Table S9:** PRISM: study population characteristics of patients from Korea and UK.
**Table S10:** PRISM: Validation results. Blue are validated results. Green on results unique to PRISM
**Table S11:** PRISM Relationship between asthma duration (years) and current biomarkers—multivariable linear regression with BH correction. Multivariable models included: age, sex, smoking status, BMI, daily oral corticosteroid dose.
**Table S12:** PRISM Biologic response after 12 months in an anti‐IL‐4Rα or anti‐IL‐5/anti‐IL‐5Rα‐treated patients.
**Table S13:** PRISM Association between sputum CXCL9 and remission score for all patients—cumulative multivariable logistic regression model: adjusted for age, sex, BMI, smoking and duration.
**Table S14:** PRISM Association between sputum CXCL9 and remission score, stratified by asthma duration (< 20 years, ≥ 20 years) ‐ cumulative multivariable logistic regression model: adjusted for age, sex, BMI, smoking and duration.
**Table S15:** Sensitivity analysis: Univariable and multivariable linear regression of associations between asthma duration and clinical, sputum, and plasma biomarkers in U‐BIOPRED, stratified by asthma onset, *adult‐onset asthma*.
**Table S16:** Sensitivity analysis: Univariable and multivariable linear regression of associations between asthma duration and clinical, sputum, and plasma biomarkers in U‐BIOPRED, stratified by asthma onset, *childhood‐onset asthma*
**Table S17:** PRISM sensitivity analysis for the association between asthma duration and sputum CXCL9 and plasma periostin by asthma age‐of‐onset.
**Table S18:** Generalised variance inflation factors (GVIF) for all covariates in the multivariable regression model of sputum CXCL9 and asthma duration, demonstrating negligible multicollinearity between all model covariates including asthma duration and age.
**Table S19:** Variance inflation factors (VIF) for asthma duration and age across all 18 sputum biomarker multivariable models in U‐BIOPRED, confirming consistently negligible multicollinearity across all models.
**Table S20:** PRISM: Collinearity diagnostics.
**Table S21:** Sensitivity analysis: Univariable and multivariable linear regression of associations between asthma duration and clinical, sputum, and plasma biomarkers in U‐BIOPRED, stratified by median age (≤ 53 years), *older cohort*.
**Table S22:** Sensitivity analysis: Univariable and multivariable linear regression of associations between asthma duration and clinical, sputum, and plasma biomarkers in U‐BIOPRED, stratified by median age (≤ 53 years), *younger cohort*.
**Table S23:** Sensitivity analysis: Univariable (ULR) and multivariable (MLR) linear regression of associations between asthma duration and clinical, sputum, and plasma biomarkers in U‐BIOPRED, restricted to participants with no prior or current biologic exposure.

## Data Availability

The data that support the findings of this study are available on request from the corresponding author. The data are not publicly available due to privacy or ethical restrictions.
