# Supplementary material for: Longer Asthma Duration Is Associated With Elevated Non‐T2 Sputum Biomarkers and Reduced T2 Inflammation in Severe Asthma
Source: Allergy. 2026 Jul 9;81(9):3286–99. doi: 10.1111/all.70436 (PMC13569612; doi:10.1111/all.70436)
Supplement: Supplementary file 1 [file ALL-81-3286-s001.docx]

**SUPPLEMENT**

**Title**

Longer asthma duration is associated with elevated non-T2 sputum biomarkers and reduced T2 inflammation in severe asthma

**Authors**

*Freda Yang^1,2^, *Sujin Seo^3^, Takehiro Hasegawa^4^, Chloe I Bloom^1^, Nazanin Zounemat-Kermani^1,5^, Pankaj K Bhavsar^1^, Katie Raby^1^, Ian M Adcock^1, 5^, ^#^Sungho Won^3,6^, ^#^Tae-Bum Kim^7^, ^#^Kian Fan Chung^1,5^ *on behalf of the* *Unbiased Biomarkers for the Prediction of Respiratory Disease Outcomes (U-BIOPRED) Consortium*

**Institutions**

1. National Heart & Lung Institute, Imperial College London, London, UK
2. Royal Brompton Hospital, Guy’s and St Thomas’ NHS Foundation Trust, London, UK
3. Institute of Health and Environment, Seoul National University, Seoul, Republic of Korea
4. Sysmex R&D Center Europe GmbH, Hamburg, Germany
5. Data Science Institute, Imperial College London, London, UK
6. Department of Public Health Sciences, Graduate School of Public Health, Seoul National University, Seoul, Republic of Korea
7. Department of Allergy and Clinical Immunology, Asan Medical Center, University of Ulsan College of Medicine, Seoul, Korea.

**Table S1.** U-BIOPRED: Number of sputum results from baseline and follow-up and total number included in study.

| Biomarker | Sputum from baseline visit | Sputum from follow-up visit | Total number of patients with sputum results |
| --- | --- | --- | --- |
| IL-6 | 191 | 45 | 236 |
| TNFa | 190 | 46 | 236 |
| TSLP | 191 | 45 | 236 |
| IL-1B | 193 | 44 | 237 |
| CCL17 | 188 | 48 | 236 |
| CCL5 | 188 | 48 | 236 |
| CCL18 | 189 | 47 | 236 |
| Periostin Total | 187 | 48 | 235 |
| Periostin Monomer | 190 | 46 | 236 |
| PAPPA | 191 | 45 | 236 |
| IL-4 | 189 | 45 | 234 |
| CCL3 | 193 | 44 | 237 |
| IL.18 | 192 | 45 | 237 |
| IL.8 | 193 | 44 | 237 |
| MPO | 193 | 44 | 237 |
| IL-17 | 190 | 46 | 236 |
| CXCL9 | 192 | 45 | 237 |
| EDN | 193 | 44 | 237 |

PAPPA: Pregnancy-Associated Plasma Protein-A, MPO: Myeloperoxidase, EDN: Eosinophil-Derived Neurotoxin

**Table S2.** U-BIOPRED: study population characteristics of patients with cytokine/mediator biomarkers and those without.

|  | **Total** | **Available** | **Not available** | **Raw P-value (adj)** |
| --- | --- | --- | --- | --- |
|  | **(N=421)** | **(N=237)** | **(N=184)** |  |
| **Age at study entry** | 54.0 [44.0, 62.0] | 55.0 [46.0, 62.0] | 52.0 [42.0, 61.0] | 0.0245 (adj: 0.184) |
| **Age at asthma diagnosis** | 26.0 [9.00, 42.0] | 27.0 [9.50, 42.0] | 22.5 [7.75, 42.0] | 0.16 (adj: 0.424) |
| Missing | 10 (2.4%) | 6 (2.5%) | 4 (2.2%) |  |
| **Duration of asthma** | 23.0 [12.0, 37.5] | 24.0 [12.0, 37.0] | 23.0 [11.0, 38.0] | 0.542 (adj: 0.781) |
| Missing | 10 (2.4%) | 6 (2.5%) | 4 (2.2%) |  |
| **Female** | 261 (62.0%) | 136 (57.4%) | 125 (67.9%) | 0.0348 (adj: 0.224) |
| **Ethnicity** |  |  |  | 0.622 (adj: 0.8) |
| Non-caucasian | 39 (9.3%) | 20 (8.4%) | 19 (10.3%) |  |
| Caucasian | 382 (90.7%) | 217 (91.6%) | 165 (89.7%) |  |
| **Smoking status** | |  |  | 0.0187 (adj: 0.168) |
| Current smoker | 42 (10.0%) | 31 (13.1%) | 11 (6.0%) |  |
| Ex smoker | 115 (27.3%) | 69 (29.1%) | 46 (25.0%) |  |
| Never smoker | 264 (62.7%) | 137 (57.8%) | 127 (69.0%) |  |
| **Smoking pack years** | 12.5 [4.00, 21.0] | 15.0 [4.88, 24.1] | 9.00 [3.10, 17.3] | 0.0693 (adj: 0.233) |
| Missing | 264 (62.7%) | 137 (57.8%) | 127 (69.0%) |  |
| **Body mass index** | 28.1 [24.6, 33.3] | 27.8 [24.6, 33.4] | 28.4 [24.8, 33.3] | 0.942 (adj: 0.963) |
| **Hay fever** | 186 (44.2%) | 104 (43.9%) | 82 (44.6%) | 0.877 (adj: 0.918) |
| Missing | 16 (3.8%) | 10 (4.2%) | 6 (3.3%) |  |
| **Allergic rhinitis** | 208 (49.4%) | 117 (49.4%) | 91 (49.5%) | 0.963 (adj: 0.963) |
| Missing | 22 (5.2%) | 13 (5.5%) | 9 (4.9%) |  |
| **Nasal polyps** | 137 (32.5%) | 86 (36.3%) | 51 (27.7%) | 0.0522 (adj: 0.226) |
| Missing | 8 (1.9%) | 2 (0.8%) | 6 (3.3%) |  |
| **Pneumonia** | 152 (36.1%) | 97 (40.9%) | 55 (29.9%) | 0.0602 (adj: 0.226) |
| Missing | 27 (6.4%) | 15 (6.3%) | 12 (6.5%) |  |
| **Vocal cord dysfunction** | 20 (4.8%) | 9 (3.8%) | 11 (6.0%) | 0.403 (adj: 0.679) |
| missing | 5 (1.2%) | 2 (0.8%) | 3 (1.6%) |  |
| **Osteoporosis** | 100 (23.8%) | 58 (24.5%) | 42 (22.8%) | 0.364 (adj: 0.679) |
| missing | 12 (2.9%) | 9 (3.8%) | 3 (1.6%) |  |
| **On daily oral steroids** | |  |  | 0.801 (adj: 0.901) |
| no | 170 (40.4%) | 96 (40.5%) | 74 (40.2%) |  |
| Previous | 34 (8.1%) | 21 (8.9%) | 13 (7.1%) |  |
| yes | 212 (50.4%) | 118 (49.8%) | 94 (51.1%) |  |
| Missing | 5 (1.2%) | 2 (0.8%) | 3 (1.6%) |  |
| **Daily oral steroid dose (prednisolone equivalent)** | 0 [0, 12.5] | 2.50 [0, 10.0] | 0 [0, 15.0] | 0.847 (adj: 0.918) |
| **Exacerbations past 12 months** | 1.00 [0, 3.00] | 1.00 [0, 3.00] | 1.00 [0, 3.00] | 0.382 (adj: 0.679) |
| Missing | 1 (0.2%) | 0 (0%) | 1 (0.5%) |  |
| **History if ICU admission** | 98 (23.3%) | 51 (21.5%) | 47 (25.5%) | 0.397 (adj: 0.679) |
| Missing | 5 (1.2%) | 4 (1.7%) | 1 (0.5%) |  |
| **History of intubation** | 41 (9.7%) | 20 (8.4%) | 21 (11.4%) | 0.432 (adj: 0.694) |
| Missing | 5 (1.2%) | 2 (0.8%) | 3 (1.6%) |  |
| **ACQ-6** | 2.17 [1.33, 3.00] | 2.00 [1.33, 2.87] | 2.33 [1.33, 3.17] | 0.254 (adj: 0.572) |
| Missing | 14 (3.3%) | 5 (2.1%) | 9 (4.9%) |  |
| **AQLQ** | 4.50 [3.53, 5.38] | 4.53 [3.66, 5.34] | 4.44 [3.47, 5.44] | 0.6 (adj: 0.794) |
| Missing | 7 (1.7%) | 4 (1.7%) | 3 (1.6%) |  |
| **SNOT** | 31.0 [18.0, 43.3] | 32.0 [21.0, 45.0] | 27.0 [16.5, 39.0] | 0.01 (adj: 0.168) |
| Missing | 9 (2.1%) | 8 (3.4%) | 1 (0.5%) |  |
| **HADS** | 12.0 [6.00, 18.0] | 12.0 [6.00, 18.0] | 11.0 [6.00, 18.0] | 0.599 (adj: 0.794) |
| Missing | 10 (2.4%) | 9 (3.8%) | 1 (0.5%) |  |
| **FeNO (ppb)** | 26.0 [15.0, 47.0] | 25.0 [14.0, 41.0] | 27.0 [15.5, 48.5] | 0.223 (adj: 0.538) |
| Missing | 27 (6.4%) | 15 (6.3%) | 12 (6.5%) |  |
| **FEV1 (% predicted)** | 66.8 [51.2, 83.5] | 65.2 [51.9, 79.9] | 68.8 [49.3, 87.2] | 0.407 (adj: 0.679) |
| Missing | 3 (0.7%) | 0 (0%) | 3 (1.6%) |  |
| **FVC (% predicted)** | 87.4 [73.9, 101] | 89.9 [76.8, 102] | 86.2 [73.2, 98.6] | 0.0551 (adj: 0.226) |
| Missing | 3 (0.7%) | 0 (0%) | 3 (1.6%) |  |
| **FEV1/FVC (%)** | 63.8 [53.1, 73.8] | 62.1 [52.7, 70.0] | 66.8 [54.7, 77.0] | <0.001 (adj: 0.0352) |
| Missing | 3 (0.7%) | 0 (0%) | 3 (1.6%) |  |
| **FEF 25-75 (% predicted)** | 29.6 [18.0, 49.1] | 27.9 [17.2, 43.2] | 34.0 [20.1, 57.5] | 0.0137 (adj: 0.168) |
| Missing | 3 (0.7%) | 0 (0%) | 3 (1.6%) |  |
| **Blood eosinophils (x10^3/uL)** | 0.200 [0.100, 0.400] | 0.200 [0.110, 0.420] | 0.200 [0.100, 0.400] | 0.0824 (adj: 0.247) |
| Missing | 13 (3.1%) | 3 (1.3%) | 10 (5.4%) |  |
| **Total IgE (IU/ml)** | 120 [47.0, 348] | 126 [51.6, 319] | 117 [44.5, 365] | 0.864 (adj: 0.918) |
| Missing | 10 (2.4%) | 3 (1.3%) | 7 (3.8%) |  |
| **Atopic** | 273 (64.8%) | 158 (66.7%) | 115 (62.5%) | 0.708 (adj: 0.862) |
| Missing | 2 (0.5%) | 1 (0.4%) | 1 (0.5%) |  |
| **hCRP mg/L** | 2.20 [0.930, 4.80] | 2.20 [0.958, 4.93] | 2.20 [0.860, 4.60] | 0.324 (adj: 0.676) |
| Missing | 8 (1.9%) | 1 (0.4%) | 7 (3.8%) |  |
| **Sputum eosinophils (%)** | 2.86 [0.430, 16.5] | 2.88 [0.390, 18.2] | 2.61 [0.655, 8.26] | 0.555 (adj: 0.781) |
| Missing | 240 (57.0%) | 87 (36.7%) | 153 (83.2%) |  |
| **Sputum neutrophils (%)** | 54.0 [34.2, 74.4] | 52.4 [32.6, 72.6] | 61.7 [49.8, 75.6] | 0.0949 (adj: 0.267) |
| Missing | 240 (57.0%) | 87 (36.7%) | 153 (83.2%) |  |

**Table S3.** U-BIOPRED Relationship between asthma duration (years) and **sputum** biomarkers – **univariable** linear regression with BH correction.

| Biomarker | n | Coefficient | CI_Lower | CI_Upper | P_value | P_adj_BH |
| --- | --- | --- | --- | --- | --- | --- |
| CCL5 | 230 | -0.858 | -2.358 | 0.643 | 0.261 | 0.428 |
| CCL17 | 230 | 1.285 | 0.017 | 2.553 | 0.047 | 0.121 |
| CCL18 | 230 | 0.851 | 0.038 | 1.663 | 0.040 | 0.121 |
| CCL3 | 231 | **1.151** | **0.351** | **1.951** | **0.005** | **0.030** |
| CXCL9 | 231 | **2.300** | **1.025** | **3.576** | **0.000** | **0.008** |
| EDN | 231 | **-1.257** | **-2.210** | **-0.304** | **0.010** | **0.045** |
| IL-1B | 231 | 0.810 | -0.787 | 2.407 | 0.319 | 0.475 |
| IL-17 | 230 | 1.046 | -7.341 | 9.433 | 0.806 | 0.806 |
| IL-18 | 231 | 2.295 | -0.784 | 5.373 | 0.143 | 0.282 |
| IL-4 | 228 | 1.124 | -5.074 | 7.322 | 0.721 | 0.764 |
| IL-6 | 230 | **2.157** | **0.883** | **3.432** | **0.001** | **0.009** |
| IL-8 | 231 | 1.140 | -0.256 | 2.537 | 0.109 | 0.245 |
| MPO | 231 | 0.575 | -0.617 | 1.768 | 0.343 | 0.475 |
| PAPPA | 230 | 3.093 | -1.196 | 7.383 | 0.157 | 0.282 |
| Periostin Monomer | 230 | 0.278 | -0.409 | 0.965 | 0.426 | 0.511 |
| Periostin Total | 229 | -0.278 | -0.911 | 0.356 | 0.389 | 0.500 |
| TNFa | 230 | 1.938 | 0.267 | 3.610 | 0.023 | 0.084 |
| TSLP | 230 | -0.826 | -3.373 | 1.721 | 0.523 | 0.589 |

Statistically significant results are highlighted in bold

**Table S4.** U-BIOPRED Relationship between asthma duration (years) and **sputum** biomarkers – **multivariable** linear regression with BH correction.

| Biomarker | n | Coefficient | CI_Lower | CI_Upper | P_value | P_adj_BH |
| --- | --- | --- | --- | --- | --- | --- |
| CCL5 | 230 | -0.006 | -0.018 | 0.006 | 0.304 | 0.421 |
| CCL17 | 230 | 0.014 | 0.00004 | 0.028 | 0.049 | 0.164 |
| CCL18 | 230 | 0.019 | -0.002 | 0.040 | 0.080 | 0.205 |
| CCL3 | 231 | 0.026 | 0.004 | 0.048 | 0.019 | 0.085 |
| CXCL9 | 231 | **0.024** | **0.011** | **0.038** | **0.001** | **0.004** |
| EDN | 231 | **-0.032** | **-0.049** | **-0.014** | **0.0005** | **0.004** |
| IL-1B | 231 | 0.007 | -0.005 | 0.018 | 0.242 | 0.410 |
| IL-17 | 230 | -0.0001 | -0.002 | 0.002 | 0.964 | 0.964 |
| IL-18 | 231 | 0.004 | -0.002 | 0.010 | 0.147 | 0.331 |
| IL-4 | 228 | 0.0004 | -0.003 | 0.003 | 0.792 | 0.838 |
| IL-6 | 230 | **0.024** | **0.010** | **0.037** | **0.001** | **0.004** |
| IL-8 | 231 | 0.008 | -0.005 | 0.021 | 0.210 | 0.410 |
| MPO | 231 | 0.005 | -0.010 | 0.020 | 0.489 | 0.550 |
| PAPPA | 230 | 0.002 | -0.002 | 0.007 | 0.250 | 0.410 |
| Periostin Monomer | 230 | 0.012 | -0.014 | 0.037 | 0.365 | 0.469 |
| Periostin Total | 229 | -0.015 | -0.044 | 0.013 | 0.297 | 0.421 |
| TNFa | 230 | 0.010 | -0.0002 | 0.021 | 0.055 | 0.164 |
| TSLP | 230 | -0.003 | -0.010 | 0.004 | 0.431 | 0.517 |

Statistically significant results are highlighted in bold

**Table S5**. U-BIOPRED Relationship between asthma duration (years) and **plasma** biomarkers – **univariable** linear regression with BH correction.

|  | n | Coefficient | CI_Lower | CI_Upper | P_value | P_adj_BH |
| --- | --- | --- | --- | --- | --- | --- |
| CCL17 | 228 | -0.638 | -2.358 | 1.082 | 0.466 | 0.770 |
| CCL18 | 229 | 1.381 | -1.874 | 4.636 | 0.404 | 0.770 |
| CCL20 | 231 | 0.964 | -1.144 | 3.073 | 0.368 | 0.770 |
| CCL27 | 229 | 2.379 | -2.493 | 7.251 | 0.337 | 0.770 |
| CCL3 | 228 | 0.836 | -3.580 | 5.251 | 0.710 | 0.786 |
| CCL5 | 229 | 0.505 | -1.816 | 2.827 | 0.668 | 0.786 |
| CXCL9 | 231 | 1.812 | -0.863 | 4.488 | 0.183 | 0.770 |
| CXCL10 | 230 | 0.590 | -2.515 | 3.694 | 0.709 | 0.786 |
| CXCL11 | 228 | 0.731 | -0.976 | 2.438 | 0.400 | 0.770 |
| EDN | 229 | -1.041 | -4.429 | 2.347 | 0.546 | 0.770 |
| IL-10 | 228 | 0.901 | -1.490 | 3.292 | 0.458 | 0.770 |
| IL-16 | 228 | -0.516 | -3.540 | 2.508 | 0.737 | 0.786 |
| IL-17 | 228 | 5.367 | -0.945 | 11.678 | 0.095 | 0.770 |
| IL-18 | 229 | -0.618 | -5.112 | 3.875 | 0.786 | 0.786 |
| IL-4 | 230 | 2.633 | -2.774 | 8.040 | 0.338 | 0.770 |
| IL-5 | 229 | -0.857 | -3.646 | 1.931 | 0.545 | 0.770 |
| IL-6 | 231 | -1.066 | -3.827 | 1.695 | 0.448 | 0.770 |
| KL-6 | 229 | 0.769 | -4.021 | 5.559 | 0.752 | 0.786 |
| MPO | 229 | -1.278 | -4.272 | 1.717 | 0.401 | 0.770 |
| PAPPA | 227 | -1.020 | -3.874 | 1.833 | 0.482 | 0.770 |
| Periostin Total | 229 | -5.032 | -10.472 | 0.407 | 0.070 | 0.770 |
| SPA | 229 | 0.773 | -4.269 | 5.815 | 0.763 | 0.786 |
| TNFa | 231 | 2.318 | -1.344 | 5.981 | 0.214 | 0.770 |
| TSLP | 227 | 0.858 | -1.245 | 2.961 | 0.422 | 0.770 |

Statistically significant results are highlighted in bold

**Table S6.** U-BIOPRED Relationship between asthma duration (years) and **plasma** biomarkers – **multivariable** linear regression with BH correction.

|  | n | Coefficient | CI_Lower | CI_Upper | P_value | P_adj_BH |
| --- | --- | --- | --- | --- | --- | --- |
| CCL17 | 228 | -0.006 | -0.017 | 0.004 | 0.258 | 0.986 |
| CCL18 | 229 | -0.0002 | -0.006 | 0.005 | 0.950 | 0.993 |
| CCL20 | 231 | 0.004 | -0.005 | 0.012 | 0.389 | 0.986 |
| CCL27 | 229 | -0.0003 | -0.004 | 0.003 | 0.858 | 0.993 |
| CCL3 | 228 | 0.001 | -0.004 | 0.005 | 0.807 | 0.993 |
| CCL5 | 229 | 0.0002 | -0.007 | 0.008 | 0.968 | 0.993 |
| CXCL9 | 231 | -0.001 | -0.007 | 0.006 | 0.866 | 0.993 |
| CXCL10 | 230 | -0.0005 | -0.006 | 0.005 | 0.864 | 0.993 |
| CXCL11 | 228 | 0.001 | -0.009 | 0.011 | 0.835 | 0.993 |
| EDN | 229 | -0.005 | -0.010 | -0.0001 | 0.047 | 0.564 |
| IL-10 | 228 | 0.001 | -0.006 | 0.009 | 0.714 | 0.993 |
| IL-16 | 228 | -0.002 | -0.008 | 0.004 | 0.590 | 0.993 |
| IL-17 | 228 | 0.002 | -0.001 | 0.005 | 0.141 | 0.844 |
| IL-18 | 229 | -0.0001 | -0.004 | 0.004 | 0.945 | 0.993 |
| IL-4 | 230 | -0.00001 | -0.003 | 0.003 | 0.993 | 0.993 |
| IL-5 | 229 | -0.003 | -0.010 | 0.003 | 0.346 | 0.986 |
| IL-6 | 231 | -0.004 | -0.010 | 0.002 | 0.227 | 0.986 |
| KL-6 | 229 | -0.0004 | -0.004 | 0.003 | 0.827 | 0.993 |
| MPO | 229 | -0.005 | -0.011 | 0.001 | 0.118 | 0.844 |
| PAPPA | 227 | -0.002 | -0.009 | 0.004 | 0.452 | 0.986 |
| Periostin Total | 229 | **-0.006** | **-0.009** | **-0.003** | **0.0001** | **0.002** |
| SPA | 229 | 0.00002 | -0.003 | 0.003 | 0.992 | 0.993 |
| TNFa | 231 | 0.002 | -0.003 | 0.007 | 0.417 | 0.986 |
| TSLP | 227 | 0.003 | -0.005 | 0.012 | 0.448 | 0.986 |

Statistically significant results are highlighted in bold

**Table S7**. U-BIOPRED Relationship between asthma duration (years) and **current** biomarkers – **univariable** linear regression with BH correction.

|  | n | Coefficient | CI_Lower | CI_Upper | P_value | P_adj_BH |
| --- | --- | --- | --- | --- | --- | --- |
| log blood eosinophil count (x10^3uL) | 398 | -8.442 | -15.980 | -0.904 | 0.028 | 0.066 |
| log blood neutrophil count (x10^3uL) | 398 | -1.163 | -5.715 | 3.389 | 0.616 | 0.616 |
| log total IgE (IU/mL) | 402 | -0.426 | -1.469 | 0.618 | 0.423 | 0.494 |
| log crp (mg/L) | 404 | 1.018 | -0.869 | 2.906 | 0.290 | 0.405 |
| log sputum eosinophil percentage (%) | 180 | **-2.537** | **-4.273** | **-0.801** | **0.004** | **0.031** |
| log sputum neutrophil percentage (%) | 180 | 3.285 | 0.484 | 6.085 | 0.022 | 0.066 |
| log FeNO (ppb) | 385 | -1.740 | -3.887 | 0.407 | 0.112 | 0.196 |

Statistically significant results are highlighted in bold.

**Table S8.** U-BIOPRED Relationship between asthma duration (years) and **current** biomarkers – **multivariable** linear regression with BH correction.

|  | n | Coefficient | CI_Lower | CI_Upper | P_value | P_adj_BH |
| --- | --- | --- | --- | --- | --- | --- |
| log blood eosinophil count (x10^3uL) | 398 | **-0.002** | **-0.003** | **-0.001** | **0.003** | **0.009** |
| log blood neutrophil count (x10^3uL) | 398 | 0.0001 | -0.002 | 0.002 | 0.962 | 0.962 |
| log total IgE (IU/mL) | 402 | -0.003 | -0.012 | 0.007 | 0.565 | 0.660 |
| log crp (mg/L) | 404 | 0.003 | -0.002 | 0.008 | 0.282 | 0.395 |
| log sputum eosinophil percentage (%) | 180 | **-0.023** | **-0.035** | **-0.011** | **0.0003** | **0.002** |
| log sputum neutrophil percentage (%) | 180 | **0.009** | **0.001** | **0.017** | **0.026** | **0.046** |
| log FeNO (ppb) | 385 | **-0.006** | **-0.010** | **-0.001** | **0.022** | **0.046** |

Statistically significant results are highlighted in bold.

**Table S9.** PRISM: study population characteristics of patients from Korea and UK.

| **Characteristic** | **Korea**, N = 424*^1^* | **UK**, N = 52*^1^* | **Overall**, N = 476*^1^* | **p-value***^2^* |
| --- | --- | --- | --- | --- |
| **Sex** |  |  |  | 0.09 |
| Male | 191 (45%) | 17 (33%) | 208 (44%) |  |
| Female | 233 (55%) | 35 (67%) | 268 (56%) |  |
| **Age, years** | 53.0 [46.0, 62.2] | 63.5 [52.0, 70.0] | 54.5 [46.0, 64.0] | <0.001 |
| **Age at diagnosis, years** | 42.0 [32.0, 52.0] | 31.0 [6.5, 46.0] | 42.0 [31.2, 51.0] | <0.001 |
| *(missing)* | 1 | 1 | 2 |  |
| **Duration** | 10.0 [5.0, 16.0] | 32.0 [20.0, 42.5] | 10.0 [5.0, 20.0] | <0.001 |
| *(missing)* | 1 | 1 | 2 |  |
| **Smoking status** |  |  |  | 0.021 |
| Never | 216 (51%) | 36 (71%) | 252 (53%) |  |
| Ex-smoker | 169 (40%) | 14 (27%) | 183 (39%) |  |
| Current | 38 (9.0%) | 1 (2.0%) | 39 (8.2%) |  |
| *(missing)* | 1 | 1 | 2 |  |
| **BMI, kg/m²** | 24.7 [22.2, 27.7] | 29.7 [23.4, 34.1] | 24.8 [22.3, 28.3] | <0.001 |
| *(missing)* | 1 | 0 | 1 |  |
| **Blood eosinophil counts, cells/uL** | 485.1 [257.8, 763.8] | 300.0 [100.0, 600.0] | 476.0 [213.6, 756.0] | 0.002 |
| *(missing)* | 0 | 3 | 3 |  |
| **Blood neutrophil counts, 10^9/L** | 4.1 [3.2, 5.3] | 4.8 [3.5, 6.2] | 4.2 [3.2, 5.4] | 0.04 |
| *(missing)* | 0 | 4 | 4 |  |
| **Sputum eosinophil, %** | 5.0 [0.0, 28.5] | 3.8 [1.5, 39.4] | 5.0 [0.0, 29.0] | 0.4 |
| *(missing)* | 109 | 31 | 140 |  |
| **Sputum neutrophil, %** | 57.5 [17.8, 84.0] | 70.3 [42.1, 79.1] | 59.0 [20.0, 82.0] | 0.4 |
| *(missing)* | 136 | 31 | 167 |  |
| **Total IgE, IU/mL** | 242.5 [95.2, 559.0] | 115.0 [50.0, 441.2] | 227.5 [84.2, 547.0] | 0.021 |
| *(missing)* | 68 | 2 | 70 |  |
| **CRP, mg/dL** | 0.2 [0.1, 0.6] | *Not available* | 0.2 [0.1, 0.6] | *Not available* |
| *(missing)* | 74 |  | 126 |  |
| **FeNO** | 53.5 [30.0, 91.2] | 38.9 [23.8, 64.8] | 52.0 [29.0, 89.0] | 0.007 |
| *(missing)* | 12 | 4 | 16 |  |
| FEV1 ,% predicted | 66.0 [51.0, 78.0] | 73.0 [59.0, 85.5] | 66.0 [52.0, 79.0] | 0.013 |
| (missing) | 2 | 1 | 3 |  |
| FEV1/FVC, % | 65.0 [57.0, 73.0] | 65.5 [59.0, 74.8] | 65.0 [57.0, 73.2] | 0.5 |
| (missing) | 2 | 2 | 4 |  |
| **Daily OCS dose, mg** | 0.0 [0.0, 3.3] | 2.0 [0.0, 10.0] | 0.0 [0.0, 4.6] | <0.001 |
| **Biologic** |  |  |  | <0.001 |
| Mepolizumab | 36 (13%) | 14 (30%) | 50 (15%) |  |
| Benralizumab | 19 (6.6%) | 32 (70%) | 51 (15%) |  |
| Dupilumab | 231 (81%) | 0 (0%) | 231 (70%) |  |
| *others* | 138 | 6 | 144 |  |

**Table S10.** PRISM: Validation results. Blue are validated results. Green on results unique to PRISM

|  |  | **Korea** | | | | **UK** | | | | **Meta-analysis** | | | | | | |  |
| --- | --- | --- | --- | --- | --- | --- | --- | --- | --- | --- | --- | --- | --- | --- | --- | --- | --- |
|  | **Analyte***^1^* | **Estimate** | **SE** | **Statistic** | **P-value** | **Estimate** | **SE** | **Statistic** | **P-value** | **Estimate** | **SE** | **Statistic** | **P-value** | **Adjusted  p-value***^2^* | **Q** | **Q p-value** | **I^2^** |
| Plasma | CCL18 | -0.007 | 0.003 | -2.065 | 0.039 | 0.002 | 0.004 | 0.427 | 0.669 | -0.003 | 0.004 | -0.668 | 0.504 | 0.606 | 2.761 | 0.097 | 64 |
| Plasma | CCL5 | -0.003 | 0.004 | -0.835 | 0.403 | 0.008 | 0.009 | 0.930 | 0.352 | 0.000 | 0.005 | -0.063 | 0.950 | 0.950 | 1.379 | 0.240 | 27 |
| Plasma | EDN | -0.001 | 0.005 | -0.192 | 0.847 | -0.010 | 0.003 | -2.852 | 0.004 | -0.006 | 0.004 | -1.511 | 0.131 | 0.338 | 1.853 | 0.173 | 46 |
| Plasma | MPO | 0.001 | 0.004 | 0.299 | 0.765 | 0.003 | 0.005 | 0.677 | 0.499 | 0.002 | 0.003 | 0.676 | 0.499 | 0.606 | 0.090 | 0.765 | 0 |
| Plasma | PAPPA | 0.002 | 0.002 | 0.770 | 0.441 | 0.008 | 0.006 | 1.354 | 0.176 | 0.002 | 0.002 | 1.074 | 0.283 | 0.439 | 1.090 | 0.296 | 8 |
| Plasma | PERIOSTIN-TOTAL | -0.003 | 0.003 | -1.185 | 0.236 | -0.006 | 0.003 | -1.902 | 0.057 | -0.004 | 0.002 | -2.132 | 0.033 | 0.128 | 0.480 | 0.488 | 0 |
| Serum | CCL17 | 0.003 | 0.003 | 0.849 | 0.396 | -0.011 | 0.004 | -2.844 | 0.004 | -0.004 | 0.007 | -0.574 | 0.566 | 0.606 | 7.381 | 0.007 | 86 |
| Serum | CCL20 | 0.052 | 0.007 | 6.893 | 0.000 | 0.003 | 0.013 | 0.266 | 0.790 | 0.029 | 0.024 | 1.194 | 0.232 | 0.400 | 10.294 | 0.001 | 90 |
| Serum | CCL3 | 0.010 | 0.007 | 1.512 | 0.131 | 0.009 | 0.006 | 1.408 | 0.159 | 0.009 | 0.005 | 2.059 | 0.040 | 0.136 | 0.030 | 0.862 | 0 |
| Serum | CXCL9 | -0.005 | 0.005 | -1.125 | 0.261 | -0.003 | 0.004 | -0.640 | 0.522 | -0.004 | 0.003 | -1.238 | 0.216 | 0.393 | 0.143 | 0.706 | 0 |
| Serum | CXCL10 | 0.000 | 0.004 | 0.125 | 0.901 | 0.007 | 0.004 | 1.781 | 0.075 | 0.004 | 0.003 | 1.093 | 0.275 | 0.439 | 1.484 | 0.223 | 33 |
| Serum | CXCL11 | -0.010 | 0.004 | -2.780 | 0.005 | 0.001 | 0.005 | 0.197 | 0.844 | -0.005 | 0.005 | -0.900 | 0.368 | 0.496 | 3.569 | 0.059 | 72 |
| Serum | IL-10 | 0.008 | 0.003 | 2.329 | 0.020 | 0.006 | 0.006 | 1.013 | 0.311 | 0.008 | 0.003 | 2.526 | 0.012 | 0.071 | 0.068 | 0.794 | 0 |
| Serum | IL-16 | -0.007 | 0.004 | -1.720 | 0.085 | -0.014 | 0.006 | -2.296 | 0.022 | -0.010 | 0.003 | -2.741 | 0.006 | **0.052** | 0.720 | 0.396 | 0 |
| Serum | IL-17 | 0.004 | 0.005 | 0.795 | 0.426 |  |  |  |  |  |  |  |  |  |  |  |  |
| Serum | IL-18 | -0.002 | 0.001 | -1.565 | 0.118 | 0.000 | 0.002 | -0.152 | 0.879 | -0.002 | 0.001 | -1.419 | 0.156 | 0.372 | 0.456 | 0.499 | 0 |
| Serum | IL-22* | -0.018 | 0.008 | -2.129 | 0.033 | 0.007 | 0.013 | 0.539 | 0.590 | -0.007 | 0.012 | -0.605 | 0.545 | 0.606 | 2.563 | 0.109 | 61 |
| Serum | IL-5 | -0.007 | 0.009 | -0.756 | 0.450 |  |  |  |  |  |  |  |  |  |  |  |  |
| Serum | IL-6 | 0.000 | 0.006 | -0.016 | 0.987 | -0.016 | 0.008 | -2.077 | 0.038 | -0.007 | 0.008 | -0.910 | 0.363 | 0.496 | 2.763 | 0.096 | 64 |
| Serum | IL-8 | 0.005 | 0.008 | 0.582 | 0.561 | 0.007 | 0.006 | 1.238 | 0.216 | 0.006 | 0.005 | 1.339 | 0.181 | 0.393 | 0.078 | 0.780 | 0 |
| Serum | KL-6* | 0.000 | 0.002 | -0.198 | 0.843 | -0.004 | 0.003 | -1.429 | 0.153 | -0.002 | 0.002 | -1.022 | 0.307 | 0.453 | 1.022 | 0.312 | 2 |
| Serum | SP-A | 0.001 | 0.002 | 0.644 | 0.519 | -0.006 | 0.003 | -2.197 | 0.028 | -0.002 | 0.003 | -0.572 | 0.567 | 0.606 | 4.492 | 0.034 | 78 |
| Serum | TNF-A | 0.004 | 0.003 | 1.520 | 0.129 | 0.004 | 0.003 | 1.735 | 0.083 | 0.004 | 0.002 | 2.306 | 0.021 | 0.109 | 0.000 | 0.990 | 0 |
| Sputum | CCL17 | 0.029 | 0.013 | 2.299 | 0.021 | 0.033 | 0.014 | 2.327 | 0.020 | 0.031 | 0.009 | 3.265 | 0.001 | **0.017** | 0.040 | 0.842 | 0 |
| Sputum | CXCL9 | 0.052 | 0.017 | 3.066 | 0.002 | 0.032 | 0.016 | 1.953 | 0.051 | 0.042 | 0.012 | 3.536 | 0.000 | 0.013 | 0.715 | 0.398 | 0 |
| Sputum | EDN | 0.013 | 0.009 | 1.495 | 0.135 | -0.009 | 0.011 | -0.827 | 0.408 | 0.003 | 0.011 | 0.271 | 0.787 | 0.813 | 2.490 | 0.115 | 60 |
| Sputum | IL-1B | 0.014 | 0.009 | 1.497 | 0.134 | 0.008 | 0.009 | 0.893 | 0.372 | 0.011 | 0.006 | 1.695 | 0.090 | 0.257 | 0.165 | 0.685 | 0 |
| Sputum | IL-18 | -0.001 | 0.002 | -0.249 | 0.804 | 0.006 | 0.004 | 1.540 | 0.123 | 0.002 | 0.003 | 0.615 | 0.539 | 0.606 | 2.102 | 0.147 | 52 |
| Sputum | IL-6 | 0.010 | 0.011 | 0.858 | 0.391 | 0.023 | 0.014 | 1.627 | 0.104 | 0.015 | 0.009 | 1.689 | 0.091 | 0.257 | 0.531 | 0.466 | 0 |
| Sputum | IL-8 | 0.006 | 0.010 | 0.559 | 0.576 | 0.030 | 0.016 | 1.910 | 0.056 | 0.015 | 0.012 | 1.273 | 0.203 | 0.393 | 1.664 | 0.197 | 40 |
| Sputum | MPO | 0.003 | 0.010 | 0.356 | 0.722 | 0.018 | 0.012 | 1.578 | 0.114 | 0.009 | 0.007 | 1.282 | 0.200 | 0.393 | 0.974 | 0.324 | 0 |
| Sputum | PERIOSTIN-MONO | 0.019 | 0.020 | 0.970 | 0.332 | 0.031 | 0.016 | 1.962 | 0.050 | 0.026 | 0.012 | 2.138 | 0.033 | 0.128 | 0.219 | 0.640 | 0 |
| Sputum | TNF-A | 0.013 | 0.006 | 2.156 | 0.031 | 0.021 | 0.012 | 1.748 | 0.080 | 0.015 | 0.005 | 2.713 | 0.007 | **0.052** | 0.343 | 0.558 | 0 |

^1^ Only Korean population has serum IL-5 and IL-17 values.

^2^ P-values are adjusted by Benjamini-Hochberg method.

^3^ Biomarkers measured in PRISM were classified as pre-specified or exploratory based on availability in U-BIOPRED. All biomarkers were pre-specified for validation in PRISM except serum IL-22 and KL-6, which were not measured in U-BIOPRED and were therefore analysed as exploratory biomarkers only.

^4^ The following biomarkers were available in U-BIOPRED but could not be assessed in PRISM: serum CCL27, CCL3, and IL-4; sputum CCL5, CCL18, CCL3, CXCL9, IL-17, IL-4, PAPPA, periostin, and TSLP.

^5^ Biomarkers highlighted in blue were significantly associated with asthma duration in U-BIOPRED and validated in PRISM. Biomarkers highlighted in green were not significantly associated with asthma duration in U-BIOPRED but reached significance in PRISM, and should be considered exploratory findings requiring further validation.

**Table S11.** PRISM Relationship between asthma duration (years) and current biomarkers – multivariable linear regression with BH correction. Multivariable models included: age, sex, smoking status, BMI, daily oral corticosteroid dose.

|  | **Analyte***^1^* | **Estimate** | **SE** | **Statistic** | **P-value** | **Adjusted  p-value***^2^* |
| --- | --- | --- | --- | --- | --- | --- |
| Clinical | Blood eosinophil counts | -6.434 | 2.435 | -2.643 | 0.008 | 0.068 |
| Clinical | Blood neutrophil counts | 0.004 | 0.007 | 0.502 | 0.616 | 0.763 |
| Clinical | total IgE | -0.871 | 2.395 | -0.364 | 0.716 | 0.818 |
| Clinical | CRP | 0.014 | 0.022 | 0.636 | 0.525 | 0.749 |
| Clinical | Sputum eosinophil | -0.269 | 0.124 | -2.164 | 0.031 | 0.113 |
| Clinical | Sputum neutrophil | 0.401 | 0.168 | 2.383 | 0.018 | 0.106 |
| Clinical | FeNO | -0.822 | 0.179 | -4.588 | 5.47E-06 | 2.19E-04 |
| *^1^* Only Korean population has CRP values. | | |  |  |  |  |
| *^2^* P-values are adjusted by Benjamini-Hochberg method. | | | |  |  |  |

**Table S12.** PRISM Biologic response after 12 months in an anti-IL-4Rα or anti-IL-5/anti-IL-5Rα-treated patients.

| **N (Korea/UK)** | **Anti_4Ra** | **Anti_IL5** | **Anti_IL5R** | **Anti_IL5/IL5R** |
| --- | --- | --- | --- | --- |
|  | **Dupilumab** | **Mepolizumab, Reslizumab** | **Benralizumab** | **Mepolizumab, Reslizumab, Benralizumab** |
|  | 257 (255/2) | 127 (88/39) | 78 (20/58) | 205 (108/97) |
| **Sputum CXCL9 measurements** | | | |  |
|  | 184 (184/0) | 65 (51/14) | 36 (17/19) | 101 (68/33) |
| **Remission at 12 month** | | | |  |
| **No exacerbations** |  |  |  |  |
| No | 46 (46/0) | 16 (12/4) | 8 (1/7) | 24 (13/11) |
| Yes | 98 (98/0) | 30 (21/9) | 20 (9/11) | 50 (30/20) |
| **mOCS ≤ 5mg** |  |  |  |  |
| No | 3 (3/0) | 1 (0/1) | 0 | 1 (0/1) |
| Yes | 141 (141/0) | 45 (33/12) | 28 (10/18) | 73 (43/30) |
| **ACQ < 1.5 or ACT > 19** |  |  |  |  |
| No | 30 (30/0) | 10 (5/5) | 4 (0/4) | 14 (5/9) |
| Yes | 114 (114/0) | 36 (28/8) | 24 (10/14) | 60 (38/22) |
| **FEV₁ ≥ 80% predicted or an improvement in FEV₁ ≥ 10%** | | |  |  |
| No | 29 (29/0) | 11 (7/4) | 4 (0/4) | 15 (7/8) |
| Yes | 113 (113/0) | 33 (24/9) | 24 (10/14) | 57 (34/33) |
| **Remission** |  |  |  |  |
| No | 75 (75/0) | 24 (17/7) | 11 (1/10) | 35 (18/17) |
| Yes | 67 (67/0) | 20 (14/6) | 17 (9/8) | 37 (23/14) |

**Table S13.** PRISM Association between sputum CXCL9 and remission score for all patients - cumulative multivariable logistic regression model: adjusted for age, sex, BMI, smoking and duration

|  | **Anti-4Ra** | | | | **Anti-IL5/IL5R** | | | |
| --- | --- | --- | --- | --- | --- | --- | --- | --- |
|  | **Estimate** | **Std. Error** | **z value** | **Pr(>\|z\|)** | **Estimate** | **Std. Error** | **z value** | **Pr(>\|z\|)** |
| age | -0.012 | 0.016 | -0.734 | 0.463 | 0.015 | 0.020 | 0.761 | 0.447 |
| sex | 0.178 | 0.516 | 0.345 | 0.730 | -0.359 | 0.579 | -0.620 | 0.535 |
| bmi | 0.016 | 0.040 | 0.410 | 0.682 | -0.026 | 0.059 | -0.449 | 0.653 |
| smoking (Ex-smoker vs Never) | -0.810 | 0.534 | -1.516 | 0.130 | 0.344 | 0.644 | 0.534 | 0.594 |
| smoking (Current vs Never) | -0.561 | 0.685 | -0.819 | 0.413 | 0.618 | 1.281 | 0.483 | 0.629 |
| duration | -0.042 | 0.020 | -2.103 | 0.036 | -0.025 | 0.021 | -1.177 | 0.239 |
| log1p(cxcl9) | -0.258 | 0.165 | -1.559 | 0.119 | 0.092 | 0.260 | 0.353 | 0.724 |
| duration:log1p(cxcl9) | 0.014 | 0.009 | 1.680 | 0.093 | -0.004 | 0.008 | -0.511 | 0.609 |
| Intercept |  |  |  |  |  |  |  |  |
| 1\|2 | -4.181 | 1.372 | -3.048 | 0.002 | -2.792 | 1.950 | -1.432 | 0.152 |
| 2\|3 | -2.687 | 1.333 | -2.015 | 0.044 | -1.829 | 1.913 | -0.956 | 0.339 |
| 3\|4 | -0.926 | 1.317 | -0.703 | 0.482 | -0.240 | 1.895 | -0.127 | 0.899 |

**Table S14.** PRISM Association between sputum CXCL9 and remission score, stratified by asthma duration (<20 years, ≥20 years) - cumulative multivariable logistic regression model: adjusted for age, sex, BMI, smoking and duration

|  | **Anti-4Ra** | | | | **Anti-IL5/IL5R** | | | |
| --- | --- | --- | --- | --- | --- | --- | --- | --- |
|  | **Estimate** | **Std. Error** | **z value** | **Pr(>\|z\|)** | **Estimate** | **Std. Error** | **z value** | **Pr(>\|z\|)** |
| age | 0.016 | 0.040 | 0.387 | 0.699 | 0.013 | 0.032 | 0.412 | 0.680 |
| sex | 0.340 | 1.180 | 0.288 | 0.773 | -0.454 | 0.832 | -0.545 | 0.586 |
| bmi | 0.001 | 0.108 | 0.010 | 0.992 | -0.128 | 0.082 | -1.573 | 0.116 |
| smoking (Ex-smoker vs Never) | -0.766 | 1.243 | -0.616 | 0.538 | 0.153 | 0.925 | 0.165 | 0.869 |
| smoking (Current vs Never) | - |  |  |  | 17.035 | 2.4.E-06 | 7.E+06 | <2e-16 |
| duration | -0.067 | 0.055 | -1.216 | 0.224 | 0.079 | 0.067 | 1.187 | 0.235 |
| log1p(cxcl9) | **-1.728** | **0.741** | **-2.333** | **0.020** | 0.803 | 0.637 | 1.261 | 0.208 |
| duration:log1p(cxcl9) | **0.066** | **0.028** | **2.380** | **0.017** | -0.025 | 0.019 | -1.331 | 0.183 |
| Intercept |  |  |  |  |  |  |  |  |
| 1\|2 | -4.198 | 3.350 | -1.253 | 0.210 | -1.961 | 3.545 | -0.553 | 0.580 |
| 2\|3 | -2.816 | 3.279 | -0.859 | 0.391 | -1.018 | 3.516 | -0.290 | 0.772 |
| 3\|4 | -0.689 | 3.251 | -0.212 | 0.832 | 0.526 | 3.510 | 0.150 | 0.881 |

Statistically significant results are highlighted in bold.

**Table S15.** Sensitivity analysis: Univariable and multivariable linear regression of associations between asthma duration and clinical, sputum, and plasma biomarkers in U-BIOPRED, stratified by asthma onset, adult-onset asthma.

| **Model** | **Biomarker (clinical)** | **N** | **Coefficient** | **CI_Lower** | **CI_Upper** | **P_value** | **P_adj_BH** |
| --- | --- | --- | --- | --- | --- | --- | --- |
| ULR | log crp (mg/L) | 249 | 0.004 | -0.005 | 0.014 | 0.350 | 0.490 |
| ULR | log total IgE (IU/mL) | 248 | -0.006 | -0.022 | 0.011 | 0.505 | 0.505 |
| ULR | log FeNO (ppb) | 239 | 0.004 | -0.004 | 0.013 | 0.341 | 0.490 |
| ULR | log blood eosinophil count (x10^3uL) | 248 | -0.001 | -0.003 | 0.002 | 0.495 | 0.505 |
| ULR | log blood neutrophil count (x10^3uL) | 248 | -0.003 | -0.006 | 0.001 | 0.125 | 0.439 |
| ULR | log sputum eosinophil percentage (%) | 113 | -0.017 | -0.040 | 0.005 | 0.123 | 0.439 |
| ULR | log sputum neutrophil percentage (%) | 113 | 0.009 | -0.006 | 0.025 | 0.233 | 0.490 |
| MLR | log crp (mg/L) | 249 | 0.006 | -0.004 | 0.016 | 0.212 | 0.503 |
| MLR | log total IgE (IU/mL) | 248 | -0.004 | -0.021 | 0.014 | 0.678 | 0.696 |
| MLR | log FeNO (ppb) | 239 | 0.002 | -0.007 | 0.011 | 0.696 | 0.696 |
| MLR | log blood eosinophil count (x10^3uL) | 248 | -0.002 | -0.004 | 0.001 | 0.244 | 0.503 |
| MLR | log blood neutrophil count (x10^3uL) | 248 | -0.002 | -0.006 | 0.002 | 0.287 | 0.503 |
| MLR | log sputum eosinophil percentage (%) | 113 | -0.025 | -0.049 | -0.001 | 0.040 | 0.282 |
| MLR | log sputum neutrophil percentage (%) | 113 | 0.006 | -0.010 | 0.022 | 0.465 | 0.650 |

| **Model** | **Biomarker (plasma)** | **N** | **Coefficient** | **CI_Lower** | **CI_Upper** | **P_value** | **P_adj_BH** |
| --- | --- | --- | --- | --- | --- | --- | --- |
| ULR | plasma_combine_CCL17 | 148 | 0.002 | -0.015 | 0.019 | 0.801 | 0.958 |
| ULR | plasma_combine_CCL18 | 149 | 0.006 | -0.003 | 0.015 | 0.167 | 0.706 |
| ULR | plasma_combine_CCL20 | 150 | 0.003 | -0.012 | 0.018 | 0.659 | 0.958 |
| ULR | plasma_combine_CCL27 | 149 | 0.004 | -0.002 | 0.011 | 0.206 | 0.706 |
| ULR | plasma_combine_CCL3 | 148 | 0.004 | -0.001 | 0.010 | 0.134 | 0.706 |
| ULR | plasma_combine_CCL5 | 149 | 0.005 | -0.009 | 0.018 | 0.523 | 0.958 |
| ULR | plasma_combine_CXCL10 | 150 | 0.002 | -0.008 | 0.012 | 0.637 | 0.958 |
| ULR | plasma_combine_CXCL11 | 148 | 0.016 | -0.002 | 0.034 | 0.085 | 0.706 |
| ULR | plasma_combine_CXCL9 | 150 | 0.013 | 0.002 | 0.024 | 0.027 | 0.641 |
| ULR | plasma_combine_EDN | 149 | -0.001 | -0.010 | 0.009 | 0.874 | 0.958 |
| ULR | plasma_combine_IL.10 | 148 | 0.002 | -0.010 | 0.014 | 0.752 | 0.958 |
| ULR | plasma_combine_IL.16 | 148 | 0.005 | -0.006 | 0.016 | 0.365 | 0.958 |
| ULR | plasma_combine_IL.17 | 148 | 0.001 | -0.003 | 0.005 | 0.608 | 0.958 |
| ULR | plasma_combine_IL.18 | 149 | -0.001 | -0.008 | 0.006 | 0.751 | 0.958 |
| ULR | plasma_combine_IL.4 | 150 | 0.001 | -0.005 | 0.006 | 0.837 | 0.958 |
| ULR | plasma_combine_IL.5 | 149 | 0.001 | -0.010 | 0.012 | 0.826 | 0.958 |
| ULR | plasma_combine_IL.6 | 150 | 0.000 | -0.011 | 0.012 | 0.958 | 0.958 |
| ULR | plasma_combine_KL.6 | 149 | 0.000 | -0.006 | 0.007 | 0.924 | 0.958 |
| ULR | plasma_combine_MPO | 149 | 0.008 | -0.004 | 0.019 | 0.204 | 0.706 |
| ULR | plasma_combine_PAPPA | 148 | -0.004 | -0.014 | 0.006 | 0.428 | 0.958 |
| ULR | plasma_combine_Periostin.total | 149 | 0.000 | -0.005 | 0.006 | 0.901 | 0.958 |
| ULR | plasma_combine_SP.A | 149 | -0.002 | -0.008 | 0.005 | 0.613 | 0.958 |
| ULR | plasma_combine_TNFa | 150 | 0.006 | -0.002 | 0.013 | 0.124 | 0.706 |
| ULR | plasma_combine_TSLP | 147 | -0.001 | -0.016 | 0.013 | 0.871 | 0.958 |
| MLR | plasma_combine_CCL17 | 148 | 0.002 | -0.017 | 0.020 | 0.873 | 0.953 |
| MLR | plasma_combine_CCL18 | 149 | 0.001 | -0.008 | 0.010 | 0.870 | 0.953 |
| MLR | plasma_combine_CCL20 | 150 | 0.005 | -0.011 | 0.021 | 0.544 | 0.953 |
| MLR | plasma_combine_CCL27 | 149 | -0.001 | -0.008 | 0.005 | 0.685 | 0.953 |
| MLR | plasma_combine_CCL3 | 148 | 0.001 | -0.005 | 0.007 | 0.699 | 0.953 |
| MLR | plasma_combine_CCL5 | 149 | 0.007 | -0.008 | 0.021 | 0.363 | 0.953 |
| MLR | plasma_combine_CXCL10 | 150 | 0.002 | -0.009 | 0.012 | 0.784 | 0.953 |
| MLR | plasma_combine_CXCL11 | 148 | 0.014 | -0.005 | 0.033 | 0.146 | 0.953 |
| MLR | plasma_combine_CXCL9 | 150 | 0.006 | -0.007 | 0.018 | 0.360 | 0.953 |
| MLR | plasma_combine_EDN | 149 | -0.006 | -0.016 | 0.004 | 0.252 | 0.953 |
| MLR | plasma_combine_IL.10 | 148 | 0.000 | -0.014 | 0.013 | 0.957 | 0.957 |
| MLR | plasma_combine_IL.16 | 148 | 0.004 | -0.008 | 0.016 | 0.479 | 0.953 |
| MLR | plasma_combine_IL.17 | 148 | 0.002 | -0.003 | 0.006 | 0.451 | 0.953 |
| MLR | plasma_combine_IL.18 | 149 | 0.002 | -0.006 | 0.009 | 0.688 | 0.953 |
| MLR | plasma_combine_IL.4 | 150 | -0.001 | -0.007 | 0.005 | 0.731 | 0.953 |
| MLR | plasma_combine_IL.5 | 149 | 0.000 | -0.013 | 0.012 | 0.955 | 0.957 |
| MLR | plasma_combine_IL.6 | 150 | -0.002 | -0.013 | 0.010 | 0.785 | 0.953 |
| MLR | plasma_combine_KL.6 | 149 | 0.001 | -0.006 | 0.008 | 0.869 | 0.953 |
| MLR | plasma_combine_MPO | 149 | 0.005 | -0.007 | 0.018 | 0.403 | 0.953 |
| MLR | plasma_combine_PAPPA | 148 | -0.003 | -0.014 | 0.008 | 0.551 | 0.953 |
| MLR | plasma_combine_Periostin.total | 149 | -0.005 | -0.010 | 0.001 | 0.091 | 0.953 |
| MLR | plasma_combine_SP.A | 149 | -0.004 | -0.009 | 0.002 | 0.217 | 0.953 |
| MLR | plasma_combine_TNFa | 150 | 0.004 | -0.003 | 0.012 | 0.267 | 0.953 |
| MLR | plasma_combine_TSLP | 147 | 0.002 | -0.014 | 0.017 | 0.856 | 0.953 |

| **Model** | **Biomarker (sputum)** | **N** | **Coefficient** | **CI_Lower** | **CI_Upper** | **P_value** | **P_adj_BH** |
| --- | --- | --- | --- | --- | --- | --- | --- |
| ULR | sputum_combine_CCL17 | 150 | 0.010 | -0.014 | 0.033 | 0.409 | 0.566 |
| ULR | sputum_combine_CCL18 | 150 | 0.039 | 0.005 | 0.072 | 0.023 | 0.070 |
| ULR | sputum_combine_CCL3 | 150 | 0.032 | -0.004 | 0.068 | 0.078 | 0.141 |
| ULR | sputum_combine_CCL5 | 150 | 0.008 | -0.010 | 0.026 | 0.386 | 0.566 |
| ULR | sputum_combine_CXCL9 | 150 | 0.022 | -0.001 | 0.044 | 0.063 | 0.141 |
| ULR | sputum_combine_EDN | 150 | -0.001 | -0.032 | 0.029 | 0.928 | 0.928 |
| ULR | sputum_combine_IL.17 | 150 | -0.001 | -0.004 | 0.003 | 0.727 | 0.786 |
| ULR | **sputum_combine_IL.18** | **150** | **0.016** | **0.006** | **0.027** | **0.002** | **0.029** |
| ULR | sputum_combine_IL.4 | 149 | 0.003 | -0.002 | 0.008 | 0.202 | 0.331 |
| ULR | sputum_combine_IL.6 | 150 | 0.027 | 0.004 | 0.051 | 0.023 | 0.070 |
| ULR | sputum_combine_IL.8 | 150 | 0.020 | -0.002 | 0.043 | 0.074 | 0.141 |
| ULR | **sputum_combine_IL1B** | **150** | **0.027** | **0.009** | **0.046** | **0.005** | **0.041** |
| ULR | sputum_combine_MPO | 150 | 0.034 | 0.009 | 0.060 | 0.009 | 0.054 |
| ULR | sputum_combine_PAPPA | 150 | 0.003 | -0.005 | 0.010 | 0.529 | 0.681 |
| ULR | sputum_combine_Periostin.Mono | 150 | 0.009 | -0.039 | 0.056 | 0.710 | 0.786 |
| ULR | sputum_combine_Periostin.total | 150 | 0.008 | -0.039 | 0.055 | 0.742 | 0.786 |
| ULR | sputum_combine_TNFa | 150 | 0.021 | 0.003 | 0.039 | 0.020 | 0.070 |
| ULR | sputum_combine_TSLP | 150 | 0.010 | -0.001 | 0.021 | 0.067 | 0.141 |
| MLR | sputum_combine_CCL17 | 150 | 0.008 | -0.017 | 0.033 | 0.530 | 0.681 |
| MLR | sputum_combine_CCL18 | 150 | 0.032 | -0.003 | 0.067 | 0.072 | 0.258 |
| MLR | sputum_combine_CCL3 | 150 | 0.020 | -0.020 | 0.059 | 0.325 | 0.542 |
| MLR | sputum_combine_CCL5 | 150 | 0.010 | -0.011 | 0.030 | 0.350 | 0.542 |
| MLR | sputum_combine_CXCL9 | 150 | 0.017 | -0.008 | 0.042 | 0.189 | 0.485 |
| MLR | sputum_combine_EDN | 150 | -0.015 | -0.048 | 0.018 | 0.362 | 0.542 |
| MLR | sputum_combine_IL.17 | 150 | -0.001 | -0.005 | 0.003 | 0.760 | 0.854 |
| MLR | sputum_combine_IL.18 | 150 | 0.017 | 0.006 | 0.029 | 0.003 | 0.052 |
| MLR | sputum_combine_IL.4 | 149 | 0.002 | -0.003 | 0.008 | 0.445 | 0.616 |
| MLR | sputum_combine_IL.6 | 150 | 0.030 | 0.004 | 0.055 | 0.023 | 0.137 |
| MLR | sputum_combine_IL.8 | 150 | 0.012 | -0.012 | 0.037 | 0.317 | 0.542 |
| MLR | sputum_combine_IL1B | 150 | 0.028 | 0.008 | 0.048 | 0.007 | 0.062 |
| MLR | sputum_combine_MPO | 150 | 0.029 | 0.002 | 0.057 | 0.036 | 0.163 |
| MLR | sputum_combine_PAPPA | 150 | 0.002 | -0.007 | 0.011 | 0.661 | 0.793 |
| MLR | sputum_combine_Periostin.Mono | 150 | 0.005 | -0.045 | 0.055 | 0.840 | 0.890 |
| MLR | sputum_combine_Periostin.total | 150 | 0.002 | -0.049 | 0.054 | 0.928 | 0.928 |
| MLR | sputum_combine_TNFa | 150 | 0.017 | -0.002 | 0.036 | 0.086 | 0.258 |
| MLR | sputum_combine_TSLP | 150 | 0.007 | -0.005 | 0.019 | 0.223 | 0.502 |

Statistically significant results are highlighted in bold.

**Table S16.** Sensitivity analysis: Univariable and multivariable linear regression of associations between asthma duration and clinical, sputum, and plasma biomarkers in U-BIOPRED, stratified by asthma onset, childhood-onset asthma

| **Model** | **Biomarker (clinical)** | **N** | **Coefficient** | **CI_Lower** | **CI_Upper** | **P_value** | **P_adj_BH** |
| --- | --- | --- | --- | --- | --- | --- | --- |
| ULR | log crp (mg/L) | 155 | 0.003 | -0.006 | 0.011 | 0.580 | 0.676 |
| ULR | log total IgE (IU/mL) | 154 | -0.004 | -0.021 | 0.013 | 0.677 | 0.677 |
| ULR | log FeNO (ppb) | 146 | -0.004 | -0.012 | 0.005 | 0.388 | 0.676 |
| ULR | log blood eosinophil count (x10^3uL) | 150 | 0.001 | -0.002 | 0.003 | 0.526 | 0.676 |
| ULR | log blood neutrophil count (x10^3uL) | 150 | -0.002 | -0.006 | 0.003 | 0.491 | 0.676 |
| ULR | log sputum eosinophil percentage (%) | 67 | 0.014 | -0.009 | 0.037 | 0.231 | 0.676 |
| ULR | log sputum neutrophil percentage (%) | 67 | 0.011 | -0.001 | 0.024 | 0.067 | 0.468 |
| MLR | log crp (mg/L) | 155 | -0.026 | -0.049 | -0.002 | 0.033 | 0.200 |
| MLR | log total IgE (IU/mL) | 154 | 0.034 | -0.015 | 0.083 | 0.171 | 0.200 |
| MLR | log FeNO (ppb) | 146 | -0.017 | -0.040 | 0.007 | 0.164 | 0.200 |
| MLR | log blood eosinophil count (x10^3uL) | 150 | -0.005 | -0.011 | 0.002 | 0.163 | 0.200 |
| MLR | log blood neutrophil count (x10^3uL) | 150 | -0.006 | -0.019 | 0.006 | 0.304 | 0.304 |
| MLR | log sputum eosinophil percentage (%) | 67 | -0.042 | -0.098 | 0.014 | 0.141 | 0.200 |
| MLR | log sputum neutrophil percentage (%) | 67 | 0.028 | -0.005 | 0.060 | 0.096 | 0.200 |

| **Model** | **Biomarker (plasma)** | **N** | **Coefficient** | **CI_Lower** | **CI_Upper** | **P_value** | **P_adj_BH** |
| --- | --- | --- | --- | --- | --- | --- | --- |
| ULR | plasma_combine_CCL17 | 80 | 0.003 | -0.019 | 0.024 | 0.814 | 0.849 |
| ULR | plasma_combine_CCL18 | 80 | 0.010 | -0.001 | 0.021 | 0.064 | 0.165 |
| ULR | plasma_combine_CCL20 | 81 | 0.015 | 0.000 | 0.029 | 0.050 | 0.165 |
| ULR | plasma_combine_CCL27 | 80 | 0.006 | -0.001 | 0.013 | 0.069 | 0.165 |
| ULR | plasma_combine_CCL3 | 80 | -0.003 | -0.012 | 0.007 | 0.574 | 0.754 |
| ULR | plasma_combine_CCL5 | 80 | 0.003 | -0.011 | 0.016 | 0.710 | 0.775 |
| ULR | plasma_combine_CXCL10 | 80 | 0.011 | 0.001 | 0.022 | 0.033 | 0.165 |
| ULR | plasma_combine_CXCL11 | 80 | 0.018 | -0.001 | 0.037 | 0.068 | 0.165 |
| ULR | **plasma_combine_CXCL9** | **81** | **0.022** | **0.012** | **0.032** | **0.000** | **0.002** |
| ULR | plasma_combine_EDN | 80 | 0.004 | -0.005 | 0.014 | 0.350 | 0.543 |
| ULR | plasma_combine_IL.10 | 80 | 0.007 | -0.008 | 0.023 | 0.362 | 0.543 |
| ULR | plasma_combine_IL.16 | 80 | 0.002 | -0.008 | 0.012 | 0.657 | 0.754 |
| ULR | plasma_combine_IL.17 | 80 | 0.004 | -0.003 | 0.010 | 0.271 | 0.487 |
| ULR | plasma_combine_IL.18 | 80 | 0.002 | -0.006 | 0.010 | 0.660 | 0.754 |
| ULR | plasma_combine_IL.4 | 80 | 0.004 | -0.003 | 0.011 | 0.269 | 0.487 |
| ULR | plasma_combine_IL.5 | 80 | -0.009 | -0.020 | 0.003 | 0.150 | 0.327 |
| ULR | plasma_combine_IL.6 | 81 | 0.003 | -0.009 | 0.015 | 0.634 | 0.754 |
| ULR | plasma_combine_KL.6 | 80 | 0.009 | 0.002 | 0.016 | 0.009 | 0.072 |
| ULR | plasma_combine_MPO | 80 | -0.004 | -0.011 | 0.003 | 0.284 | 0.487 |
| ULR | plasma_combine_PAPPA | 79 | 0.001 | -0.012 | 0.014 | 0.865 | 0.865 |
| ULR | plasma_combine_Periostin.total | 80 | 0.002 | -0.004 | 0.007 | 0.556 | 0.754 |
| ULR | **plasma_combine_SP.A** | **80** | **0.010** | **0.004** | **0.016** | **0.002** | **0.022** |
| ULR | plasma_combine_TNFa | 81 | 0.010 | -0.001 | 0.020 | 0.066 | 0.165 |
| ULR | plasma_combine_TSLP | 80 | 0.016 | 0.000 | 0.032 | 0.050 | 0.165 |
| MLR | plasma_combine_CCL17 | 80 | -0.048 | -0.116 | 0.019 | 0.159 | 0.506 |
| MLR | plasma_combine_CCL18 | 80 | 0.005 | -0.028 | 0.038 | 0.760 | 0.793 |
| MLR | plasma_combine_CCL20 | 81 | -0.032 | -0.076 | 0.013 | 0.159 | 0.506 |
| MLR | plasma_combine_CCL27 | 80 | 0.008 | -0.013 | 0.030 | 0.436 | 0.615 |
| MLR | plasma_combine_CCL3 | 80 | -0.015 | -0.045 | 0.016 | 0.348 | 0.597 |
| MLR | **plasma_combine_CCL5** | **80** | **-0.061** | **-0.098** | **-0.024** | **0.002** | **0.019** |
| MLR | plasma_combine_CXCL10 | 80 | -0.013 | -0.045 | 0.019 | 0.409 | 0.614 |
| MLR | plasma_combine_CXCL11 | 80 | -0.034 | -0.093 | 0.026 | 0.259 | 0.564 |
| MLR | plasma_combine_CXCL9 | 81 | -0.011 | -0.042 | 0.021 | 0.500 | 0.667 |
| MLR | plasma_combine_EDN | 80 | -0.014 | -0.044 | 0.015 | 0.342 | 0.597 |
| MLR | plasma_combine_IL.10 | 80 | 0.035 | -0.015 | 0.086 | 0.169 | 0.506 |
| MLR | plasma_combine_IL.16 | 80 | -0.016 | -0.047 | 0.016 | 0.335 | 0.597 |
| MLR | plasma_combine_IL.17 | 80 | -0.004 | -0.026 | 0.018 | 0.737 | 0.793 |
| MLR | plasma_combine_IL.18 | 80 | -0.015 | -0.039 | 0.010 | 0.239 | 0.564 |
| MLR | plasma_combine_IL.4 | 80 | 0.005 | -0.017 | 0.027 | 0.678 | 0.775 |
| MLR | plasma_combine_IL.5 | 80 | -0.029 | -0.066 | 0.009 | 0.137 | 0.506 |
| MLR | plasma_combine_IL.6 | 81 | -0.044 | -0.079 | -0.009 | 0.014 | 0.115 |
| MLR | plasma_combine_KL.6 | 80 | -0.005 | -0.027 | 0.016 | 0.613 | 0.767 |
| MLR | plasma_combine_MPO | 80 | -0.015 | -0.037 | 0.008 | 0.194 | 0.518 |
| MLR | **plasma_combine_PAPPA** | **79** | **-0.076** | **-0.113** | **-0.038** | **0.000** | **0.002** |
| MLR | plasma_combine_Periostin.total | 80 | -0.007 | -0.025 | 0.010 | 0.398 | 0.614 |
| MLR | plasma_combine_SP.A | 80 | 0.004 | -0.014 | 0.023 | 0.639 | 0.767 |
| MLR | plasma_combine_TNFa | 81 | 0.026 | -0.008 | 0.060 | 0.134 | 0.506 |
| MLR | plasma_combine_TSLP | 80 | 0.007 | -0.045 | 0.059 | 0.795 | 0.795 |

| **Model** | **Biomarker** | **N** | **Coefficient** | **CI_Lower** | **CI_Upper** | **P_value** | **P_adj_BH** |
| --- | --- | --- | --- | --- | --- | --- | --- |
| ULR | sputum_combine_CCL17 | 80 | 0.027 | 0.001 | 0.054 | 0.043 | 0.361 |
| ULR | sputum_combine_CCL18 | 80 | 0.044 | -0.002 | 0.091 | 0.060 | 0.361 |
| ULR | sputum_combine_CCL3 | 81 | 0.031 | -0.011 | 0.073 | 0.149 | 0.446 |
| ULR | sputum_combine_CCL5 | 80 | 0.001 | -0.025 | 0.026 | 0.968 | 0.968 |
| ULR | sputum_combine_CXCL9 | 81 | 0.021 | -0.004 | 0.046 | 0.100 | 0.446 |
| ULR | sputum_combine_EDN | 81 | 0.008 | -0.024 | 0.040 | 0.627 | 0.822 |
| ULR | sputum_combine_IL.17 | 80 | 0.001 | -0.003 | 0.005 | 0.532 | 0.798 |
| ULR | sputum_combine_IL.18 | 81 | -0.001 | -0.010 | 0.009 | 0.895 | 0.966 |
| ULR | sputum_combine_IL.4 | 79 | -0.002 | -0.007 | 0.003 | 0.503 | 0.798 |
| ULR | sputum_combine_IL.6 | 80 | 0.019 | -0.005 | 0.043 | 0.126 | 0.446 |
| ULR | sputum_combine_IL.8 | 81 | 0.009 | -0.013 | 0.031 | 0.437 | 0.798 |
| ULR | sputum_combine_IL1B | 81 | -0.008 | -0.028 | 0.013 | 0.461 | 0.798 |
| ULR | sputum_combine_MPO | 81 | 0.002 | -0.025 | 0.028 | 0.912 | 0.966 |
| ULR | sputum_combine_PAPPA | 80 | 0.006 | 0.001 | 0.012 | 0.023 | 0.361 |
| ULR | sputum_combine_Periostin.Mono | 80 | 0.010 | -0.033 | 0.052 | 0.648 | 0.822 |
| ULR | sputum_combine_Periostin.total | 79 | -0.012 | -0.068 | 0.045 | 0.685 | 0.822 |
| ULR | sputum_combine_TNFa | 80 | 0.008 | -0.011 | 0.028 | 0.401 | 0.798 |
| ULR | sputum_combine_TSLP | 80 | -0.009 | -0.024 | 0.006 | 0.248 | 0.637 |
| MLR | sputum_combine_CCL17 | 80 | 0.054 | -0.033 | 0.141 | 0.218 | 0.976 |
| MLR | sputum_combine_CCL18 | 80 | 0.089 | -0.057 | 0.235 | 0.229 | 0.976 |
| MLR | sputum_combine_CCL3 | 81 | 0.004 | -0.132 | 0.140 | 0.956 | 0.976 |
| MLR | sputum_combine_CCL5 | 80 | 0.001 | -0.082 | 0.085 | 0.976 | 0.976 |
| MLR | sputum_combine_CXCL9 | 81 | 0.022 | -0.056 | 0.101 | 0.574 | 0.976 |
| MLR | sputum_combine_EDN | 81 | -0.036 | -0.140 | 0.067 | 0.484 | 0.976 |
| MLR | sputum_combine_IL.17 | 80 | 0.006 | -0.007 | 0.019 | 0.379 | 0.976 |
| MLR | sputum_combine_IL.18 | 81 | 0.004 | -0.027 | 0.036 | 0.785 | 0.976 |
| MLR | sputum_combine_IL.4 | 79 | -0.002 | -0.018 | 0.015 | 0.847 | 0.976 |
| MLR | sputum_combine_IL.6 | 80 | 0.036 | -0.042 | 0.113 | 0.360 | 0.976 |
| MLR | sputum_combine_IL.8 | 81 | -0.010 | -0.083 | 0.063 | 0.786 | 0.976 |
| MLR | sputum_combine_IL1B | 81 | -0.013 | -0.081 | 0.055 | 0.711 | 0.976 |
| MLR | sputum_combine_MPO | 81 | -0.026 | -0.114 | 0.061 | 0.551 | 0.976 |
| MLR | sputum_combine_PAPPA | 80 | 0.004 | -0.013 | 0.021 | 0.612 | 0.976 |
| MLR | sputum_combine_Periostin.Mono | 80 | -0.004 | -0.135 | 0.127 | 0.951 | 0.976 |
| MLR | sputum_combine_Periostin.total | 79 | -0.063 | -0.237 | 0.111 | 0.470 | 0.976 |
| MLR | sputum_combine_TNFa | 80 | -0.011 | -0.075 | 0.053 | 0.732 | 0.976 |
| MLR | sputum_combine_TSLP | 80 | -0.021 | -0.070 | 0.029 | 0.409 | 0.976 |

Statistically significant results are highlighted in bold.

**Table S17.** PRISM sensitivity analysis for the association between asthma duration and sputum CXCL9 and plasma periostin by asthma age-of-onset

|  |  |  | **Korea** | | | | **UK** | | | | **Meta-analysis** | | | | | | | | |
| --- | --- | --- | --- | --- | --- | --- | --- | --- | --- | --- | --- | --- | --- | --- | --- | --- | --- | --- | --- |
| **Model^1^** | **Term** | **AIC** | **Estimate** | **SE** | **Statistic** | **P** | **Estimate** | **SE** | **Statistic** | **P** | **Estimate** | **SE** | **CI_L** | **CI_U** | **Statistic** | **P-** | **Q** | **Q p** | **I^2^** |
| **Sputum CXCL9** | | | | | | | | | | | | | | | | | | | |
| Null | duration | 1755.4 | 0.052 | 0.017 | 3.066 | 0.002 | 0.032 | 0.016 | 1.953 | 0.051 | 0.042 | 0.012 | 0.019 | 0.065 | 3.536 | 4.1E-4 | 0.715 | 0.398 | 0 |
| Covariate | duration | 1755.7 | 0.065 | 0.018 | 3.544 | 3.9E-4 | 0.046 | 0.031 | 1.488 | 0.137 | 0.060 | 0.016 | 0.029 | 0.090 | 3.805 | 1.4E-4 | 0.292 | 0.589 | 0 |
| Interaction | duration | 1757.2 | -0.048 | 0.117 | -0.411 | 0.681 | -0.002 | 0.048 | -0.043 | 0.966 | -0.009 | 0.045 | -0.096 | 0.079 | -0.197 | 0.844 | 0.132 | 0.717 | 0 |
|  | interaction |  | 0.115 | 0.117 | 0.980 | 0.327 | 0.062 | 0.049 | 1.264 | 0.206 | 0.070 | 0.046 | -0.019 | 0.160 | 1.545 | 0.122 | 0.170 | 0.680 | 0 |
| Startification | Childhood | 1752.1 | 1.430 | 1.999 | 0.715 | 0.474 | 0.076 | 0.044 | 1.731 | 0.083 | 0.076 | 0.044 | -0.009 | 0.162 | 1.746 | 0.081 | 0.459 | 0.498 | 0 |
|  | Adult |  | 0.068 | 0.018 | 3.656 | 2.6E-4 | 0.041 | 0.036 | 1.153 | 0.249 | 0.062 | 0.016 | 0.030 | 0.094 | 3.777 | 1.6E-4 | 0.430 | 0.512 | 0 |
| **Plasma periostin** | | | | | | | | | | | | | | | | | | | |
| Null | duration | 912.45 | -0.003 | 0.003 | -1.185 | 0.236 | -0.006 | 0.003 | -1.902 | 0.057 | -0.004 | 0.002 | -0.008 | -3.3E-4 | -2.132 | 0.033 | 0.480 | 0.488 | 0 |
| Covariate | duration | 914.3 | -0.004 | 0.003 | -1.340 | 0.180 | -0.007 | 0.005 | -1.452 | 0.146 | -0.005 | 0.002 | -0.009 | 2.0E-4 | -1.880 | 0.060 | 0.371 | 0.542 | 0 |
| Interaction | duration | 912.6 | 2.5E-4 | 0.010 | 0.025 | 0.980 | -0.023 | 0.009 | -2.480 | 0.013 | -0.012 | 0.011 | -0.034 | 0.011 | -1.014 | 0.311 | 2.833 | 0.092 | 64.706 |
|  | interaction |  | -0.004 | 0.010 | -0.416 | 0.677 | 0.017 | 0.009 | 1.975 | 0.048 | 0.007 | 0.011 | -0.014 | 0.028 | 0.668 | 0.504 | 2.529 | 0.112 | 60.461 |
| Startification | Childhood | 937.5 | -0.063 | 0.039 | -1.616 | 0.106 | 0.032 | 0.014 | 2.307 | 0.021 | -0.009 | 0.047 | -0.100 | 0.083 | -0.183 | 0.855 | 5.261 | 0.022 | 80.992 |
|  | Adult |  | -0.004 | 0.003 | -1.389 | 0.165 | -0.004 | 0.015 | -0.281 | 0.778 | -0.004 | 0.003 | -0.010 | 0.002 | -1.417 | 0.156 | 0.000 | 0.990 | 0 |

^1^ Childhood-onset : <18 years, adult-onset : $\geq$18 years

**Table S18.** Generalised variance inflation factors (GVIF) for all covariates in the multivariable regression model of sputum CXCL9 and asthma duration, demonstrating negligible multicollinearity between all model covariates including asthma duration and age.

| **Variable** | **GVIF** | **Df** | **GVIF^(1/(2*Df))** |
| --- | --- | --- | --- |
| Asthma duration | 1.14 | 1 | 1.07 |
| Age | 1.12 | 1 | 1.06 |
| Sex | 1.06 | 1 | 1.03 |
| Race | 1.03 | 1 | 1.01 |
| Smoking status | 1.15 | 2 | 1.04 |
| BMI | 1.07 | 1 | 1.03 |
| Oral corticosteroid dose | 1.05 | 1 | 1.03 |

**Table S19.** Variance inflation factors (VIF) for asthma duration and age across all 18 sputum biomarker multivariable models in U-BIOPRED, confirming consistently negligible multicollinearity across all models.

| **Biomarker** | **VIF_duration** | **VIF_age** |
| --- | --- | --- |
| sputum_combine_IL.6 | 1.14 | 1.12 |
| sputum_combine_TNFa | 1.14 | 1.12 |
| sputum_combine_TSLP | 1.14 | 1.12 |
| sputum_combine_IL1B | 1.14 | 1.12 |
| sputum_combine_CCL17 | 1.14 | 1.12 |
| sputum_combine_CCL5 | 1.14 | 1.12 |
| sputum_combine_CCL18 | 1.14 | 1.12 |
| sputum_combine_Periostin.total | 1.14 | 1.12 |
| sputum_combine_Periostin.Mono | 1.14 | 1.12 |
| sputum_combine_PAPPA | 1.13 | 1.11 |
| sputum_combine_IL.4 | 1.13 | 1.12 |
| sputum_combine_CCL3 | 1.14 | 1.12 |
| sputum_combine_IL.18 | 1.14 | 1.12 |
| sputum_combine_IL.8 | 1.14 | 1.12 |
| sputum_combine_MPO | 1.14 | 1.12 |
| sputum_combine_IL.17 | 1.14 | 1.12 |
| sputum_combine_CXCL9 | 1.14 | 1.12 |
| sputum_combine_EDN | 1.14 | 1.12 |

**Table S20.** PRISM: Collinearity diagnostics

| **Term** | **VIF** | **Increased SE** | **Tolerance** |
| --- | --- | --- | --- |
| KOREA |  |  |  |
| duration | 1.190 | 1.090 | 0.840 |
| age | 1.280 | 1.130 | 0.780 |
| sex | 2.000 | 1.420 | 0.500 |
| smoking | 2.350 | 1.530 | 0.430 |
| bmi | 1.120 | 1.060 | 0.890 |
| ocs_daily | 1.050 | 1.020 | 0.950 |
| UK |  |  |  |
| duration | 1.190 | 1.090 | 0.840 |
| age | 1.630 | 1.280 | 0.610 |
| sex | 1.590 | 1.260 | 0.630 |
| smoking | 1.030 | 1.020 | 0.970 |
| bmi | 1.310 | 1.150 | 0.760 |
| ocs_daily | 1.790 | 1.340 | 0.560 |

**Table S21.** Sensitivity analysis: Univariable and multivariable linear regression of associations between asthma duration and clinical, sputum, and plasma biomarkers in U-BIOPRED, stratified by median age (≤53 years), older cohort.

| **Model** | **Biomarker (clinical)** | **N** | **Coefficient** | **CI_Lower** | **CI_Upper** | **P_value** | **P_adj_BH** |
| --- | --- | --- | --- | --- | --- | --- | --- |
| ULR | log crp (mg/L) | 202 | 0.004 | -0.003 | 0.010 | 0.292 | 0.511 |
| ULR | log total IgE (IU/mL) | 200 | 0.000 | -0.011 | 0.012 | 0.956 | 0.965 |
| ULR | log FeNO (ppb) | 195 | -0.004 | -0.010 | 0.001 | 0.132 | 0.307 |
| ULR | log blood eosinophil count (x10^3uL) | 199 | -0.002 | -0.004 | 0.000 | 0.035 | 0.123 |
| ULR | log blood neutrophil count (x10^3uL) | 199 | 0.000 | -0.003 | 0.003 | 0.965 | 0.965 |
| ULR | log sputum eosinophil percentage (%) | **101** | **-0.021** | **-0.035** | **-0.007** | **0.004** | **0.027** |
| ULR | log sputum neutrophil percentage (%) | 101 | 0.002 | -0.007 | 0.010 | 0.696 | 0.965 |
| MLR | log crp (mg/L) | 202 | 0.004 | -0.003 | 0.011 | 0.230 | 0.402 |
| MLR | log total IgE (IU/mL) | 200 | 0.000 | -0.012 | 0.012 | 0.952 | 0.952 |
| MLR | log FeNO (ppb) | 195 | -0.004 | -0.010 | 0.001 | 0.142 | 0.331 |
| MLR | log blood eosinophil count (x10^3uL) | 199 | -0.002 | -0.004 | 0.000 | 0.036 | 0.126 |
| MLR | log blood neutrophil count (x10^3uL) | 199 | 0.000 | -0.002 | 0.003 | 0.742 | 0.875 |
| MLR | log sputum eosinophil percentage (%) | **101** | **-0.026** | **-0.041** | **-0.011** | **0.001** | **0.004** |
| MLR | log sputum neutrophil percentage (%) | 101 | 0.002 | -0.008 | 0.011 | 0.750 | 0.875 |

| **Model** | **Biomarker (plasma)** | **N** | **Coefficient** | **CI_Lower** | **CI_Upper** | **P_value** | **P_adj_BH** |
| --- | --- | --- | --- | --- | --- | --- | --- |
| ULR | plasma_combine_CCL17 | 124 | -0.005 | -0.018 | 0.007 | 0.402 | 0.728 |
| ULR | plasma_combine_CCL18 | 124 | -0.001 | -0.007 | 0.006 | 0.871 | 0.909 |
| ULR | plasma_combine_CCL20 | 125 | 0.006 | -0.004 | 0.016 | 0.263 | 0.628 |
| ULR | plasma_combine_CCL27 | 124 | -0.001 | -0.006 | 0.003 | 0.551 | 0.728 |
| ULR | plasma_combine_CCL3 | 124 | -0.005 | -0.009 | 0.000 | 0.058 | 0.495 |
| ULR | plasma_combine_CCL5 | 124 | 0.005 | -0.004 | 0.014 | 0.288 | 0.628 |
| ULR | plasma_combine_CXCL10 | 125 | 0.001 | -0.005 | 0.007 | 0.780 | 0.869 |
| ULR | plasma_combine_CXCL11 | 124 | 0.005 | -0.007 | 0.017 | 0.445 | 0.728 |
| ULR | plasma_combine_CXCL9 | 125 | 0.003 | -0.005 | 0.011 | 0.463 | 0.728 |
| ULR | plasma_combine_EDN | 124 | -0.004 | -0.010 | 0.002 | 0.218 | 0.626 |
| ULR | plasma_combine_IL.10 | 124 | 0.001 | -0.009 | 0.011 | 0.797 | 0.869 |
| ULR | plasma_combine_IL.16 | 124 | -0.006 | -0.013 | 0.001 | 0.068 | 0.495 |
| ULR | plasma_combine_IL.17 | 122 | 0.003 | 0.000 | 0.006 | 0.083 | 0.495 |
| ULR | plasma_combine_IL.18 | 124 | 0.002 | -0.004 | 0.007 | 0.561 | 0.728 |
| ULR | plasma_combine_IL.4 | 125 | 0.001 | -0.003 | 0.005 | 0.607 | 0.728 |
| ULR | plasma_combine_IL.5 | 124 | -0.005 | -0.012 | 0.003 | 0.203 | 0.626 |
| ULR | plasma_combine_IL.6 | 125 | 0.000 | -0.007 | 0.007 | 0.983 | 0.983 |
| ULR | plasma_combine_KL.6 | 124 | 0.003 | -0.001 | 0.007 | 0.175 | 0.626 |
| ULR | plasma_combine_MPO | 124 | -0.004 | -0.012 | 0.003 | 0.224 | 0.626 |
| ULR | plasma_combine_PAPPA | 123 | -0.003 | -0.010 | 0.005 | 0.463 | 0.728 |
| ULR | plasma_combine_Periostin.total | 124 | -0.005 | -0.008 | -0.001 | 0.014 | 0.343 |
| ULR | plasma_combine_SP.A | 124 | -0.002 | -0.006 | 0.003 | 0.493 | 0.728 |
| ULR | plasma_combine_TNFa | 125 | 0.001 | -0.004 | 0.006 | 0.592 | 0.728 |
| ULR | plasma_combine_TSLP | 122 | 0.006 | -0.004 | 0.017 | 0.235 | 0.626 |
| MLR | plasma_combine_CCL17 | 124 | -0.007 | -0.020 | 0.006 | 0.283 | 0.616 |
| MLR | plasma_combine_CCL18 | 124 | -0.003 | -0.010 | 0.003 | 0.323 | 0.647 |
| MLR | plasma_combine_CCL20 | 125 | 0.006 | -0.004 | 0.017 | 0.240 | 0.577 |
| MLR | plasma_combine_CCL27 | 124 | -0.002 | -0.006 | 0.003 | 0.395 | 0.655 |
| MLR | plasma_combine_CCL3 | 124 | -0.005 | -0.010 | 0.000 | 0.054 | 0.495 |
| MLR | plasma_combine_CCL5 | 124 | 0.004 | -0.005 | 0.013 | 0.361 | 0.655 |
| MLR | plasma_combine_CXCL10 | 125 | 0.002 | -0.005 | 0.008 | 0.649 | 0.778 |
| MLR | plasma_combine_CXCL11 | 124 | 0.005 | -0.007 | 0.017 | 0.424 | 0.655 |
| MLR | plasma_combine_CXCL9 | 125 | 0.003 | -0.005 | 0.010 | 0.504 | 0.712 |
| MLR | plasma_combine_EDN | 124 | -0.006 | -0.013 | 0.001 | 0.081 | 0.495 |
| MLR | plasma_combine_IL.10 | 124 | 0.001 | -0.010 | 0.011 | 0.918 | 0.962 |
| MLR | plasma_combine_IL.16 | 124 | -0.006 | -0.013 | 0.002 | 0.124 | 0.495 |
| MLR | plasma_combine_IL.17 | 122 | 0.003 | -0.001 | 0.006 | 0.119 | 0.495 |
| MLR | plasma_combine_IL.18 | 124 | 0.002 | -0.003 | 0.008 | 0.437 | 0.655 |
| MLR | plasma_combine_IL.4 | 125 | 0.000 | -0.005 | 0.004 | 0.943 | 0.962 |
| MLR | plasma_combine_IL.5 | 124 | -0.007 | -0.014 | 0.001 | 0.094 | 0.495 |
| MLR | plasma_combine_IL.6 | 125 | -0.001 | -0.008 | 0.006 | 0.708 | 0.809 |
| MLR | plasma_combine_KL.6 | 124 | 0.003 | -0.002 | 0.008 | 0.193 | 0.577 |
| MLR | plasma_combine_MPO | 124 | -0.005 | -0.013 | 0.002 | 0.170 | 0.577 |
| MLR | plasma_combine_PAPPA | 123 | -0.002 | -0.010 | 0.006 | 0.615 | 0.777 |
| MLR | **plasma_combine_Periostin.total** | **124** | **-0.006** | **-0.010** | **-0.002** | **0.001** | **0.034** |
| MLR | plasma_combine_SP.A | 124 | 0.000 | -0.004 | 0.004 | 0.962 | 0.962 |
| MLR | plasma_combine_TNFa | 125 | 0.001 | -0.004 | 0.006 | 0.608 | 0.777 |
| MLR | plasma_combine_TSLP | 122 | 0.007 | -0.004 | 0.018 | 0.231 | 0.577 |

| **Model** | **Biomarker (sputum)** | **N** | **Coefficient** | **CI_Lower** | **CI_Upper** | **P_value** | **P_adj_BH** |
| --- | --- | --- | --- | --- | --- | --- | --- |
| ULR | sputum_combine_CCL17 | 125 | 0.004 | -0.012 | 0.020 | 0.614 | 0.721 |
| ULR | sputum_combine_CCL18 | 125 | 0.007 | -0.016 | 0.029 | 0.555 | 0.721 |
| ULR | sputum_combine_CCL3 | 125 | 0.016 | -0.009 | 0.041 | 0.204 | 0.523 |
| ULR | sputum_combine_CCL5 | 125 | -0.011 | -0.024 | 0.003 | 0.122 | 0.523 |
| ULR | sputum_combine_CXCL9 | 125 | 0.014 | -0.002 | 0.030 | 0.094 | 0.523 |
| ULR | **sputum_combine_EDN** | **125** | **-0.036** | **-0.056** | **-0.016** | **0.001** | **0.009** |
| ULR | sputum_combine_IL.17 | 125 | 0.000 | -0.003 | 0.003 | 0.999 | 0.999 |
| ULR | sputum_combine_IL.18 | 125 | 0.003 | -0.004 | 0.010 | 0.433 | 0.721 |
| ULR | sputum_combine_IL.4 | 123 | -0.001 | -0.004 | 0.003 | 0.641 | 0.721 |
| ULR | sputum_combine_IL.6 | 125 | 0.018 | 0.002 | 0.034 | 0.029 | 0.262 |
| ULR | sputum_combine_IL.8 | 125 | 0.006 | -0.009 | 0.021 | 0.455 | 0.721 |
| ULR | sputum_combine_IL1B | 125 | 0.006 | -0.008 | 0.019 | 0.431 | 0.721 |
| ULR | sputum_combine_MPO | 125 | 0.007 | -0.012 | 0.025 | 0.486 | 0.721 |
| ULR | sputum_combine_PAPPA | 124 | 0.001 | -0.003 | 0.005 | 0.610 | 0.721 |
| ULR | sputum_combine_Periostin.Mono | 125 | -0.005 | -0.038 | 0.028 | 0.762 | 0.807 |
| ULR | sputum_combine_Periostin.total | 125 | -0.022 | -0.054 | 0.009 | 0.166 | 0.523 |
| ULR | sputum_combine_TNFa | 125 | 0.009 | -0.004 | 0.022 | 0.183 | 0.523 |
| ULR | sputum_combine_TSLP | 125 | -0.002 | -0.009 | 0.005 | 0.557 | 0.721 |
| MLR | sputum_combine_CCL17 | 125 | 0.005 | -0.012 | 0.022 | 0.546 | 0.717 |
| MLR | sputum_combine_CCL18 | 125 | 0.010 | -0.014 | 0.033 | 0.427 | 0.717 |
| MLR | sputum_combine_CCL3 | 125 | 0.014 | -0.012 | 0.040 | 0.298 | 0.712 |
| MLR | sputum_combine_CCL5 | 125 | -0.007 | -0.021 | 0.007 | 0.309 | 0.712 |
| MLR | sputum_combine_CXCL9 | 125 | 0.014 | -0.003 | 0.032 | 0.104 | 0.468 |
| MLR | **sputum_combine_EDN** | **125** | **-0.042** | **-0.062** | **-0.021** | **0.000** | **0.002** |
| MLR | sputum_combine_IL.17 | 125 | 0.000 | -0.003 | 0.003 | 0.934 | 0.934 |
| MLR | sputum_combine_IL.18 | 125 | 0.004 | -0.004 | 0.011 | 0.356 | 0.712 |
| MLR | sputum_combine_IL.4 | 123 | -0.001 | -0.005 | 0.002 | 0.502 | 0.717 |
| MLR | sputum_combine_IL.6 | 125 | 0.021 | 0.004 | 0.038 | 0.017 | 0.155 |
| MLR | sputum_combine_IL.8 | 125 | 0.004 | -0.012 | 0.020 | 0.608 | 0.729 |
| MLR | sputum_combine_IL1B | 125 | 0.005 | -0.009 | 0.020 | 0.459 | 0.717 |
| MLR | sputum_combine_MPO | 125 | 0.006 | -0.013 | 0.025 | 0.558 | 0.717 |
| MLR | sputum_combine_PAPPA | 124 | 0.001 | -0.004 | 0.005 | 0.711 | 0.786 |
| MLR | sputum_combine_Periostin.Mono | 125 | -0.005 | -0.037 | 0.027 | 0.743 | 0.786 |
| MLR | sputum_combine_Periostin.total | 125 | -0.028 | -0.061 | 0.005 | 0.097 | 0.468 |
| MLR | sputum_combine_TNFa | 125 | 0.007 | -0.007 | 0.020 | 0.320 | 0.712 |
| MLR | sputum_combine_TSLP | 125 | -0.005 | -0.012 | 0.003 | 0.238 | 0.712 |

Statistically significant results are highlighted in bold.

**Table S22.** Sensitivity analysis: Univariable and multivariable linear regression of associations between asthma duration and clinical, sputum, and plasma biomarkers in U-BIOPRED, stratified by median age (≤53 years), younger cohort.

| **Model** | **Biomarker (clinical)** | **N** | **Coefficient** | **CI_Lower** | **CI_Upper** | **P_value** | **P_adj_BH** |
| --- | --- | --- | --- | --- | --- | --- | --- |
| ULR | log crp (mg/L) | 202 | 0.001 | -0.007 | 0.009 | 0.817 | 0.817 |
| ULR | log total IgE (IU/mL) | 202 | -0.010 | -0.026 | 0.006 | 0.207 | 0.458 |
| ULR | log FeNO (ppb) | 190 | -0.004 | -0.013 | 0.004 | 0.327 | 0.458 |
| ULR | log blood eosinophil count (x10^3uL) | 199 | -0.001 | -0.003 | 0.001 | 0.188 | 0.458 |
| ULR | log blood neutrophil count (x10^3uL) | 199 | -0.001 | -0.005 | 0.003 | 0.528 | 0.616 |
| ULR | log sputum eosinophil percentage (%) | 79 | -0.012 | -0.035 | 0.011 | 0.288 | 0.458 |
| ULR | **log sputum neutrophil percentage (%)** | **79** | **0.024** | **0.010** | **0.039** | **0.001** | **0.009** |
| MLR | log crp (mg/L) | 202 | -0.003 | -0.012 | 0.005 | 0.413 | 0.578 |
| MLR | log total IgE (IU/mL) | 202 | -0.004 | -0.021 | 0.013 | 0.638 | 0.638 |
| MLR | log FeNO (ppb) | 190 | -0.004 | -0.012 | 0.005 | 0.400 | 0.578 |
| MLR | log blood eosinophil count (x10^3uL) | 199 | -0.002 | -0.004 | 0.000 | 0.090 | 0.211 |
| MLR | log blood neutrophil count (x10^3uL) | 199 | -0.001 | -0.005 | 0.003 | 0.542 | 0.633 |
| MLR | log sputum eosinophil percentage (%) | 79 | -0.022 | -0.044 | 0.001 | 0.057 | 0.199 |
| MLR | **log sputum neutrophil percentage (%)** | **79** | **0.024** | **0.008** | **0.040** | **0.004** | **0.027** |

| **Model** | **Biomarker (plasma)** | **N** | **Coefficient** | **CI_Lower** | **CI_Upper** | **P_value** | **P_adj_BH** |
| --- | --- | --- | --- | --- | --- | --- | --- |
| ULR | plasma_combine_CCL17 | 104 | -0.001 | -0.019 | 0.017 | 0.931 | 0.979 |
| ULR | plasma_combine_CCL18 | 105 | 0.004 | -0.005 | 0.014 | 0.351 | 0.828 |
| ULR | plasma_combine_CCL20 | 106 | -0.002 | -0.016 | 0.012 | 0.780 | 0.979 |
| ULR | plasma_combine_CCL27 | 105 | 0.006 | 0.000 | 0.012 | 0.067 | 0.738 |
| ULR | plasma_combine_CCL3 | 104 | 0.010 | 0.003 | 0.017 | 0.004 | 0.094 |
| ULR | plasma_combine_CCL5 | 105 | -0.006 | -0.019 | 0.007 | 0.387 | 0.828 |
| ULR | plasma_combine_CXCL10 | 105 | -0.001 | -0.011 | 0.010 | 0.858 | 0.979 |
| ULR | plasma_combine_CXCL11 | 104 | -0.003 | -0.021 | 0.015 | 0.753 | 0.979 |
| ULR | plasma_combine_CXCL9 | 106 | 0.000 | -0.010 | 0.011 | 0.938 | 0.979 |
| ULR | plasma_combine_EDN | 105 | 0.001 | -0.008 | 0.010 | 0.867 | 0.979 |
| ULR | plasma_combine_IL.10 | 104 | 0.004 | -0.006 | 0.014 | 0.449 | 0.828 |
| ULR | plasma_combine_IL.16 | 104 | 0.009 | -0.001 | 0.019 | 0.092 | 0.738 |
| ULR | plasma_combine_IL.17 | 106 | 0.001 | -0.004 | 0.006 | 0.645 | 0.979 |
| ULR | plasma_combine_IL.18 | 105 | -0.004 | -0.010 | 0.002 | 0.196 | 0.828 |
| ULR | plasma_combine_IL.4 | 105 | 0.002 | -0.003 | 0.007 | 0.388 | 0.828 |
| ULR | plasma_combine_IL.5 | 105 | 0.005 | -0.007 | 0.016 | 0.416 | 0.828 |
| ULR | plasma_combine_IL.6 | 106 | -0.008 | -0.020 | 0.004 | 0.175 | 0.828 |
| ULR | plasma_combine_KL.6 | 105 | -0.004 | -0.010 | 0.003 | 0.257 | 0.828 |
| ULR | plasma_combine_MPO | 105 | 0.000 | -0.010 | 0.010 | 0.980 | 0.980 |
| ULR | plasma_combine_PAPPA | 104 | -0.001 | -0.012 | 0.010 | 0.884 | 0.979 |
| ULR | plasma_combine_Periostin.total | 105 | -0.002 | -0.008 | 0.003 | 0.403 | 0.828 |
| ULR | plasma_combine_SP.A | 105 | 0.002 | -0.004 | 0.007 | 0.488 | 0.837 |
| ULR | plasma_combine_TNFa | 106 | 0.006 | -0.004 | 0.015 | 0.250 | 0.828 |
| ULR | plasma_combine_TSLP | 105 | -0.002 | -0.016 | 0.013 | 0.827 | 0.979 |
| MLR | plasma_combine_CCL17 | 104 | -0.004 | -0.024 | 0.016 | 0.690 | 0.790 |
| MLR | plasma_combine_CCL18 | 105 | 0.000 | -0.010 | 0.009 | 0.928 | 0.928 |
| MLR | plasma_combine_CCL20 | 106 | -0.006 | -0.021 | 0.009 | 0.452 | 0.727 |
| MLR | plasma_combine_CCL27 | 105 | 0.004 | -0.002 | 0.011 | 0.162 | 0.648 |
| MLR | plasma_combine_CCL3 | 104 | 0.011 | 0.003 | 0.018 | 0.007 | 0.156 |
| MLR | plasma_combine_CCL5 | 105 | -0.006 | -0.020 | 0.009 | 0.436 | 0.727 |
| MLR | plasma_combine_CXCL10 | 105 | -0.007 | -0.018 | 0.004 | 0.200 | 0.672 |
| MLR | plasma_combine_CXCL11 | 104 | -0.004 | -0.023 | 0.016 | 0.724 | 0.790 |
| MLR | plasma_combine_CXCL9 | 106 | -0.004 | -0.015 | 0.007 | 0.482 | 0.727 |
| MLR | plasma_combine_EDN | 105 | -0.002 | -0.011 | 0.007 | 0.589 | 0.788 |
| MLR | plasma_combine_IL.10 | 104 | 0.003 | -0.009 | 0.014 | 0.645 | 0.790 |
| MLR | plasma_combine_IL.16 | 104 | 0.006 | -0.005 | 0.017 | 0.280 | 0.672 |
| MLR | plasma_combine_IL.17 | 106 | 0.001 | -0.005 | 0.006 | 0.762 | 0.795 |
| MLR | plasma_combine_IL.18 | 105 | -0.006 | -0.012 | 0.000 | 0.059 | 0.341 |
| MLR | plasma_combine_IL.4 | 105 | -0.001 | -0.006 | 0.004 | 0.699 | 0.790 |
| MLR | plasma_combine_IL.5 | 105 | 0.004 | -0.008 | 0.017 | 0.471 | 0.727 |
| MLR | plasma_combine_IL.6 | 106 | -0.013 | -0.026 | 0.000 | 0.054 | 0.341 |
| MLR | plasma_combine_KL.6 | 105 | -0.008 | -0.014 | -0.001 | 0.022 | 0.260 |
| MLR | plasma_combine_MPO | 105 | -0.004 | -0.014 | 0.007 | 0.485 | 0.727 |
| MLR | plasma_combine_PAPPA | 104 | -0.006 | -0.018 | 0.005 | 0.280 | 0.672 |
| MLR | plasma_combine_Periostin.total | 105 | -0.005 | -0.011 | 0.001 | 0.071 | 0.341 |
| MLR | plasma_combine_SP.A | 105 | 0.003 | -0.002 | 0.008 | 0.243 | 0.672 |
| MLR | plasma_combine_TNFa | 106 | 0.003 | -0.008 | 0.013 | 0.591 | 0.788 |
| MLR | plasma_combine_TSLP | 105 | -0.006 | -0.021 | 0.010 | 0.457 | 0.727 |

| **Model** | **Biomarker (sputum)** | **N** | **Coefficient** | **CI_Lower** | **CI_Upper** | **P_value** | **P_adj_BH** |
| --- | --- | --- | --- | --- | --- | --- | --- |
| ULR | sputum_combine_CCL17 | 105 | 0.028 | 0.005 | 0.052 | 0.018 | 0.079 |
| ULR | sputum_combine_CCL18 | 105 | 0.040 | 0.000 | 0.080 | 0.049 | 0.176 |
| ULR | sputum_combine_CCL3 | 106 | 0.052 | 0.015 | 0.088 | 0.006 | 0.055 |
| ULR | sputum_combine_CCL5 | 105 | -0.002 | -0.023 | 0.019 | 0.844 | 0.893 |
| ULR | **sputum_combine_CXCL9** | **106** | **0.040** | **0.019** | **0.060** | **0.000** | **0.005** |
| ULR | sputum_combine_EDN | 106 | -0.005 | -0.037 | 0.027 | 0.749 | 0.842 |
| ULR | sputum_combine_IL.17 | 105 | 0.000 | -0.002 | 0.003 | 0.715 | 0.842 |
| ULR | sputum_combine_IL.18 | 106 | 0.006 | -0.004 | 0.015 | 0.224 | 0.367 |
| ULR | sputum_combine_IL.4 | 105 | 0.004 | -0.001 | 0.008 | 0.145 | 0.290 |
| ULR | sputum_combine_IL.6 | 105 | 0.028 | 0.006 | 0.050 | 0.013 | 0.076 |
| ULR | sputum_combine_IL.8 | 106 | 0.017 | -0.004 | 0.038 | 0.118 | 0.266 |
| ULR | sputum_combine_IL1B | 106 | 0.006 | -0.012 | 0.023 | 0.514 | 0.771 |
| ULR | sputum_combine_MPO | 106 | 0.006 | -0.018 | 0.029 | 0.645 | 0.830 |
| ULR | sputum_combine_PAPPA | 106 | 0.005 | -0.003 | 0.013 | 0.182 | 0.328 |
| ULR | sputum_combine_Periostin.Mono | 105 | 0.037 | -0.003 | 0.077 | 0.068 | 0.205 |
| ULR | sputum_combine_Periostin.total | 104 | 0.002 | -0.048 | 0.052 | 0.930 | 0.930 |
| ULR | sputum_combine_TNFa | 105 | 0.014 | -0.002 | 0.031 | 0.088 | 0.225 |
| ULR | sputum_combine_TSLP | 105 | -0.004 | -0.017 | 0.010 | 0.564 | 0.781 |
| MLR | sputum_combine_CCL17 | 105 | 0.026 | 0.001 | 0.051 | 0.046 | 0.207 |
| MLR | sputum_combine_CCL18 | 105 | 0.033 | -0.010 | 0.075 | 0.129 | 0.386 |
| MLR | sputum_combine_CCL3 | 106 | 0.041 | 0.003 | 0.079 | 0.035 | 0.207 |
| MLR | sputum_combine_CCL5 | 105 | -0.008 | -0.030 | 0.015 | 0.503 | 0.646 |
| MLR | **sputum_combine_CXCL9** | **106** | **0.038** | **0.016** | **0.059** | **0.001** | **0.013** |
| MLR | sputum_combine_EDN | 106 | -0.018 | -0.051 | 0.015 | 0.276 | 0.452 |
| MLR | sputum_combine_IL.17 | 105 | 0.000 | -0.003 | 0.003 | 0.967 | 0.993 |
| MLR | sputum_combine_IL.18 | 106 | 0.006 | -0.004 | 0.017 | 0.214 | 0.452 |
| MLR | sputum_combine_IL.4 | 105 | 0.003 | -0.002 | 0.008 | 0.263 | 0.452 |
| MLR | sputum_combine_IL.6 | 105 | 0.024 | 0.001 | 0.048 | 0.042 | 0.207 |
| MLR | sputum_combine_IL.8 | 106 | 0.011 | -0.011 | 0.034 | 0.326 | 0.488 |
| MLR | sputum_combine_IL1B | 106 | 0.008 | -0.012 | 0.027 | 0.440 | 0.609 |
| MLR | sputum_combine_MPO | 106 | 0.000 | -0.026 | 0.026 | 0.993 | 0.993 |
| MLR | sputum_combine_PAPPA | 106 | 0.005 | -0.004 | 0.013 | 0.276 | 0.452 |
| MLR | sputum_combine_Periostin.Mono | 105 | 0.038 | -0.004 | 0.080 | 0.076 | 0.272 |
| MLR | sputum_combine_Periostin.total | 104 | 0.008 | -0.046 | 0.063 | 0.768 | 0.922 |
| MLR | sputum_combine_TNFa | 105 | 0.012 | -0.006 | 0.029 | 0.187 | 0.452 |
| MLR | sputum_combine_TSLP | 105 | 0.000 | -0.014 | 0.015 | 0.978 | 0.993 |

Statistically significant results are highlighted in bold.

**Table S23.** Sensitivity analysis: Univariable (ULR) and multivariable (MLR) linear regression of associations between asthma duration and clinical, sputum, and plasma biomarkers in U-BIOPRED, restricted to participants with no prior or current biologic exposure.

| **Model** | **Biomarker** | **N** | **Coefficient** | **CI_Lower** | **CI_Upper** | **p** | **P_adj_BH** |
| --- | --- | --- | --- | --- | --- | --- | --- |
| **Clinical biomarkers** | | | | | | | |
| ULR | log crp (mg/L) | 347 | 0.004 | -0.002 | 0.009 | 0.202 | 0.283 |
| ULR | log total IgE (IU/mL) | 346 | -0.001 | -0.011 | 0.009 | 0.910 | 0.910 |
| ULR | log FeNO (ppb) | 331 | -0.005 | -0.010 | 0.000 | 0.057 | 0.099 |
| ULR | log blood eosinophil count (x10^3uL) | 345 | -0.001 | -0.003 | 0.000 | 0.043 | 0.099 |
| ULR | log blood neutrophil count (x10^3uL) | 345 | -0.001 | -0.003 | 0.001 | 0.443 | 0.517 |
| ULR | log sputum eosinophil percentage (%) | 165 | -0.016 | -0.028 | -0.004 | 0.011 | 0.077 |
| ULR | log sputum neutrophil percentage (%) | 165 | 0.009 | 0.001 | 0.017 | 0.027 | 0.095 |
| MLR | log crp (mg/L) | 347 | 0.002 | -0.003 | 0.007 | 0.460 | 0.644 |
| MLR | log total IgE (IU/mL) | 346 | -0.001 | -0.011 | 0.010 | 0.869 | 0.869 |
| MLR | log FeNO (ppb) | 331 | -0.005 | -0.010 | 0.000 | 0.060 | 0.104 |
| MLR | log blood eosinophil count (x10^3uL) | 345 | -0.002 | -0.003 | 0.000 | 0.015 | 0.053 |
| MLR | log blood neutrophil count (x10^3uL) | 345 | -0.001 | -0.003 | 0.002 | 0.579 | 0.676 |
| MLR | **log sputum eosinophil percentage (%)** | **165** | **-0.022** | **-0.034** | **-0.010** | **0.001** | **0.004** |
| MLR | log sputum neutrophil percentage (%) | 165 | 0.009 | 0.001 | 0.017 | 0.038 | 0.089 |
| **Plasma biomarkers** | | | | | | | |
| ULR | plasma_combine_CCL17 | 200 | -0.006 | -0.017 | 0.004 | 0.231 | 0.851 |
| ULR | plasma_combine_CCL18 | 201 | 0.004 | -0.002 | 0.009 | 0.219 | 0.851 |
| ULR | plasma_combine_CCL20 | 203 | 0.002 | -0.007 | 0.010 | 0.667 | 0.873 |
| ULR | plasma_combine_CCL27 | 201 | 0.001 | -0.003 | 0.005 | 0.709 | 0.873 |
| ULR | plasma_combine_CCL3 | 200 | 0.002 | -0.002 | 0.006 | 0.341 | 0.851 |
| ULR | plasma_combine_CCL5 | 201 | 0.001 | -0.007 | 0.008 | 0.903 | 0.919 |
| ULR | plasma_combine_CXCL10 | 202 | 0.001 | -0.005 | 0.007 | 0.711 | 0.873 |
| ULR | plasma_combine_CXCL11 | 200 | 0.005 | -0.006 | 0.016 | 0.354 | 0.851 |
| ULR | plasma_combine_CXCL9 | 203 | 0.005 | -0.002 | 0.012 | 0.146 | 0.851 |
| ULR | plasma_combine_EDN | 201 | 0.000 | -0.006 | 0.005 | 0.900 | 0.919 |
| ULR | plasma_combine_IL.10 | 200 | 0.002 | -0.005 | 0.010 | 0.568 | 0.873 |
| ULR | plasma_combine_IL.16 | 200 | -0.001 | -0.008 | 0.005 | 0.672 | 0.873 |
| ULR | plasma_combine_IL.17 | 200 | 0.003 | 0.000 | 0.006 | 0.073 | 0.851 |
| ULR | plasma_combine_IL.18 | 201 | -0.001 | -0.005 | 0.003 | 0.741 | 0.873 |
| ULR | plasma_combine_IL.4 | 202 | 0.002 | -0.002 | 0.005 | 0.330 | 0.851 |
| ULR | plasma_combine_IL.5 | 201 | -0.002 | -0.008 | 0.005 | 0.604 | 0.873 |
| ULR | plasma_combine_IL.6 | 203 | -0.003 | -0.009 | 0.004 | 0.436 | 0.872 |
| ULR | plasma_combine_KL.6 | 201 | 0.001 | -0.003 | 0.004 | 0.764 | 0.873 |
| ULR | plasma_combine_MPO | 201 | -0.003 | -0.009 | 0.004 | 0.423 | 0.872 |
| ULR | plasma_combine_PAPPA | 199 | -0.002 | -0.009 | 0.005 | 0.570 | 0.873 |
| ULR | plasma_combine_Periostin.total | 201 | -0.003 | -0.006 | 0.001 | 0.150 | 0.851 |
| ULR | plasma_combine_SP.A | 201 | 0.000 | -0.003 | 0.004 | 0.919 | 0.919 |
| ULR | plasma_combine_TNFa | 203 | 0.003 | -0.002 | 0.008 | 0.230 | 0.851 |
| ULR | plasma_combine_TSLP | 199 | 0.004 | -0.005 | 0.013 | 0.354 | 0.851 |
| MLR | plasma_combine_CCL17 | 200 | -0.007 | -0.018 | 0.004 | 0.201 | 0.920 |
| MLR | plasma_combine_CCL18 | 201 | 0.000 | -0.006 | 0.006 | 0.958 | 0.996 |
| MLR | plasma_combine_CCL20 | 203 | 0.001 | -0.008 | 0.010 | 0.782 | 0.996 |
| MLR | plasma_combine_CCL27 | 201 | -0.001 | -0.005 | 0.002 | 0.499 | 0.996 |
| MLR | plasma_combine_CCL3 | 200 | 0.001 | -0.003 | 0.005 | 0.651 | 0.996 |
| MLR | plasma_combine_CCL5 | 201 | 0.000 | -0.008 | 0.008 | 0.966 | 0.996 |
| MLR | plasma_combine_CXCL10 | 202 | -0.001 | -0.007 | 0.005 | 0.774 | 0.996 |
| MLR | plasma_combine_CXCL11 | 200 | 0.003 | -0.008 | 0.014 | 0.638 | 0.996 |
| MLR | plasma_combine_CXCL9 | 203 | 0.001 | -0.005 | 0.008 | 0.678 | 0.996 |
| MLR | plasma_combine_EDN | 201 | -0.003 | -0.009 | 0.002 | 0.230 | 0.920 |
| MLR | plasma_combine_IL.10 | 200 | 0.001 | -0.007 | 0.009 | 0.794 | 0.996 |
| MLR | plasma_combine_IL.16 | 200 | -0.002 | -0.009 | 0.005 | 0.576 | 0.996 |
| MLR | plasma_combine_IL.17 | 200 | 0.003 | 0.000 | 0.006 | 0.072 | 0.866 |
| MLR | plasma_combine_IL.18 | 201 | 0.000 | -0.005 | 0.004 | 0.840 | 0.996 |
| MLR | plasma_combine_IL.4 | 202 | 0.000 | -0.004 | 0.004 | 0.932 | 0.996 |
| MLR | plasma_combine_IL.5 | 201 | -0.003 | -0.010 | 0.004 | 0.367 | 0.996 |
| MLR | plasma_combine_IL.6 | 203 | -0.005 | -0.012 | 0.001 | 0.121 | 0.920 |
| MLR | plasma_combine_KL.6 | 201 | 0.000 | -0.004 | 0.004 | 0.996 | 0.996 |
| MLR | plasma_combine_MPO | 201 | -0.005 | -0.012 | 0.002 | 0.156 | 0.920 |
| MLR | plasma_combine_PAPPA | 199 | -0.002 | -0.008 | 0.005 | 0.660 | 0.996 |
| MLR | **plasma_combine_Periostin.total** | **201** | **-0.005** | **-0.009** | **-0.002** | **0.002** | **0.043** |
| MLR | plasma_combine_SP.A | 201 | 0.000 | -0.003 | 0.004 | 0.884 | 0.996 |
| MLR | plasma_combine_TNFa | 203 | 0.002 | -0.004 | 0.008 | 0.475 | 0.996 |
| MLR | plasma_combine_TSLP | 199 | 0.004 | -0.006 | 0.013 | 0.436 | 0.996 |
| **Sputum biomarkers** | | | | | | | |
| ULR | sputum_combine_CCL17 | 202 | 0.012 | -0.002 | 0.026 | 0.100 | 0.225 |
| ULR | sputum_combine_CCL18 | 202 | 0.023 | 0.001 | 0.045 | 0.039 | 0.142 |
| ULR | **sputum_combine_CCL3** | **203** | **0.035** | **0.013** | **0.056** | **0.002** | **0.019** |
| ULR | sputum_combine_CCL5 | 202 | 0.001 | -0.011 | 0.012 | 0.911 | 0.911 |
| ULR | **sputum_combine_CXCL9** | **203** | **0.022** | **0.008** | **0.036** | **0.002** | **0.019** |
| ULR | sputum_combine_EDN | 203 | -0.018 | -0.037 | 0.001 | 0.065 | 0.167 |
| ULR | sputum_combine_IL.17 | 202 | 0.000 | -0.002 | 0.003 | 0.835 | 0.884 |
| ULR | sputum_combine_IL.18 | 203 | 0.003 | -0.003 | 0.009 | 0.347 | 0.481 |
| ULR | sputum_combine_IL.4 | 201 | 0.001 | -0.002 | 0.004 | 0.606 | 0.727 |
| ULR | **sputum_combine_IL.6** | **202** | **0.020** | **0.006** | **0.034** | **0.004** | **0.025** |
| ULR | sputum_combine_IL.8 | 203 | 0.013 | 0.000 | 0.025 | 0.054 | 0.161 |
| ULR | sputum_combine_IL1B | 203 | 0.006 | -0.005 | 0.017 | 0.289 | 0.452 |
| ULR | sputum_combine_MPO | 203 | 0.009 | -0.006 | 0.024 | 0.263 | 0.452 |
| ULR | sputum_combine_PAPPA | 203 | 0.003 | -0.002 | 0.007 | 0.214 | 0.429 |
| ULR | sputum_combine_Periostin.Mono | 202 | 0.014 | -0.012 | 0.040 | 0.301 | 0.452 |
| ULR | sputum_combine_Periostin.total | 201 | -0.008 | -0.037 | 0.021 | 0.576 | 0.727 |
| ULR | sputum_combine_TNFa | 202 | 0.012 | 0.001 | 0.022 | 0.037 | 0.142 |
| ULR | sputum_combine_TSLP | 202 | -0.001 | -0.008 | 0.006 | 0.827 | 0.884 |
| MLR | sputum_combine_CCL17 | 202 | 0.011 | -0.004 | 0.026 | 0.148 | 0.333 |
| MLR | sputum_combine_CCL18 | 202 | 0.018 | -0.005 | 0.040 | 0.129 | 0.333 |
| MLR | **sputum_combine_CCL3** | **203** | **0.031** | **0.008** | **0.054** | **0.010** | **0.043** |
| MLR | sputum_combine_CCL5 | 202 | 0.001 | -0.011 | 0.013 | 0.870 | 0.921 |
| MLR | **sputum_combine_CXCL9** | **203** | **0.024** | **0.009** | **0.039** | **0.002** | **0.023** |
| MLR | **sputum_combine_EDN** | **203** | **-0.028** | **-0.047** | **-0.009** | **0.004** | **0.023** |
| MLR | sputum_combine_IL.17 | 202 | 0.000 | -0.002 | 0.003 | 0.985 | 0.985 |
| MLR | sputum_combine_IL.18 | 203 | 0.003 | -0.004 | 0.009 | 0.439 | 0.592 |
| MLR | sputum_combine_IL.4 | 201 | 0.001 | -0.003 | 0.004 | 0.689 | 0.775 |
| MLR | **sputum_combine_IL.6** | **202** | **0.021** | **0.007** | **0.036** | **0.004** | **0.023** |
| MLR | sputum_combine_IL.8 | 203 | 0.010 | -0.004 | 0.024 | 0.143 | 0.333 |
| MLR | sputum_combine_IL1B | 203 | 0.007 | -0.005 | 0.018 | 0.275 | 0.496 |
| MLR | sputum_combine_MPO | 203 | 0.006 | -0.010 | 0.022 | 0.460 | 0.592 |
| MLR | sputum_combine_PAPPA | 203 | 0.003 | -0.002 | 0.007 | 0.266 | 0.496 |
| MLR | sputum_combine_Periostin.Mono | 202 | 0.012 | -0.015 | 0.038 | 0.384 | 0.575 |
| MLR | sputum_combine_Periostin.total | 201 | -0.016 | -0.046 | 0.014 | 0.303 | 0.496 |
| MLR | sputum_combine_TNFa | 202 | 0.009 | -0.002 | 0.021 | 0.113 | 0.333 |
| MLR | sputum_combine_TSLP | 202 | -0.002 | -0.009 | 0.006 | 0.645 | 0.774 |

Statistically significant results are highlighted in bold.
